# Nemo-like kinase disrupts nuclear import and drives TDP43 mislocalization in ALS

**DOI:** 10.1172/JCI188138

**Published:** 2025-06-24

**Authors:** Michael E. Bekier, Emile Pinarbasi, Gopinath Krishnan, Jack J. Mesojedec, Madelaine Hurley, Harisankar Harikumar Sheela, Catherine A. Collins, Layla Ghaffari, Martina de Majo, Erik M. Ullian, Mark Koontz, Sarah Coleman, Xingli Li, Elizabeth M.H. Tank, Jacob Waksmacki, Fen-Biao Gao, Sami J. Barmada

**Affiliations:** 1 Department of Neurology and; 2 Department of Pathology, Michigan Medicine, University of Michigan, Ann Arbor, Michigan, USA.; 3 Frontotemporal Dementia Research Center, RNA Therapeutics Institute, University of Massachusetts Chan Medical School, Worcester, Massachusetts, USA.; 4 Department of Molecular, Cellular, and Developmental Biology, University of Michigan, Ann Arbor, Michigan, USA.; 5 Department of Neurosciences, Case Western Reserve University School of Medicine, Cleveland, Ohio, USA.; 6 Synapticure, Chicago, Illinois, USA.; 7 Department of Ophthalmology, University of California, San Francisco, San Francisco, California, USA.

**Keywords:** Cell biology, Neuroscience, ALS, Molecular pathology, Transport

## Abstract

Cytoplasmic transactive response DNA-binding protein 43 (TDP43) mislocalization and aggregation are pathological hallmarks of amyotrophic lateral sclerosis (ALS). However, the initial cellular insults that lead to TDP43 mislocalization remain unclear. In this study, we demonstrate that nemo-like kinase (NLK) — a proline-directed serine-threonine kinase — promotes the mislocalization of TDP43 and other RNA-binding proteins by disrupting nuclear import. NLK levels were selectively elevated in neurons exhibiting TDP43 mislocalization in tissues from patients with ALS, and genetic reduction of *NLK* reduced toxicity in human neuron models of ALS. Our findings suggest that NLK is a promising therapeutic target for neurodegenerative diseases.

## Abstract 2

Introduces a novel mechanism for nucleocytoplasmic transport defects in TDP43 proteinopathies and provides a framework for targeting NLK in neurodegenerative diseases.

## Introduction

Amyotrophic lateral sclerosis (ALS) is a fatal and progressive neurodegenerative disease distinguished by loss of motor neurons in the cortex and spinal cord (1). The clinical presentation of ALS is heterogeneous, depending in large part upon the neuroanatomical pathways involved. Despite this, a single pathological hallmark — mislocalization and aggregation of the RNA-binding protein transactive response DNA-binding protein 43 (TDP43) — is found in more than 95% of individuals with ALS (2). TDP43 is crucial for several aspects of RNA processing, including RNA splicing, stability, and transport (3–6). TDP43 is primarily nuclear in healthy cells, in contrast to the nuclear exclusion and cytoplasmic deposition of TDP43 that are characteristic of ALS (2). Although the origins of TDP43 mislocalization are unclear, deficiencies in nucleocytoplasmic transport machinery are increasingly implicated as a potential contributing factor to ALS pathology (7). Impaired nuclear import of TDP43 and other RNA-binding proteins, together with dysfunction of the nuclear pore complex itself, are observed in human induced pluripotent stem cell–derived (iPSC-derived) neurons from patients with sporadic or familial ALS as well as postmortem samples (8–10). Genetic screens in ALS model systems have repeatedly uncovered nuclear pore and nucleocytoplasmic transport factors as potent disease modifiers, confirming the significance of these pathways in disease pathogenesis (10–12).

Among the most consistent and dramatic disease modifiers to emerge from these screens is nemo-like kinase (NLK), a proline-directed serine-threonine kinase that regulates cell differentiation, proliferation, and apoptosis (13–15). NLK depletion mitigates disease phenotypes not only in *Drosophila* and murine models of ALS but also in murine models of spinobulbar muscular atrophy and spinocerebellar ataxia (16–18). Conversely, NLK overexpression reduces toxicity in Huntington’s disease models, indicating context-dependent effects of NLK in neurodegeneration (19).

NLK has several predicted substrates that function in nucleocytoplasmic transport, but whether NLK itself may influence nuclear import and ALS pathogenesis remains unknown. Here, we demonstrate that NLK is a pivotal regulator of nucleocytoplasmic transport. NLK upregulation leads directly to cytoplasmic accumulation of TDP43 and other RNA-binding proteins associated with ALS, and reduction in NLK promotes the survival of iPSC-derived neurons carrying ALS-associated mutations. Importantly, NLK is upregulated selectively in affected neurons from patients with ALS and correlates with TDP43 mislocalization, implicating NLK as a key determinant of disease pathophysiology and highlighting NLK as a promising therapeutic target for ALS and related TDP43 proteinopathies.

## Results

### NLK overexpression drives mislocalization of TDP43 and other ALS-linked RNA-binding proteins.

**Figure 1:**
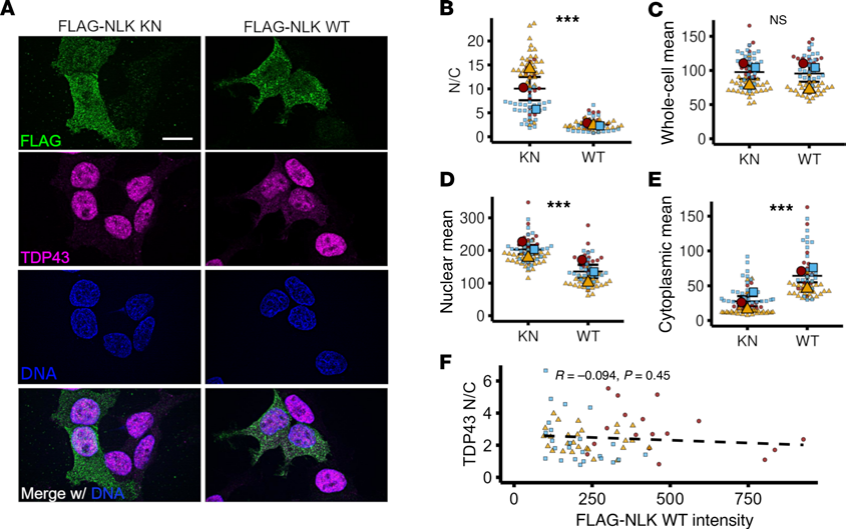
Overexpression of NLK leads to cytoplasmic accumulation of TDP43. (**A**) HEK293T cells were transfected with plasmids encoding either FLAG-NLK-KN or FLAG-NLK-WT, followed by immunofluorescence using antibodies against FLAG (green) and TDP43 (magenta); DNA was stained with Hoechst (blue). Scale bar: 10 μm. (**B**–**E**) Superplots showing the nuclear/cytoplasmic (N/C) ratio (**B**), whole-cell intensity (**C**), nuclear intensity (**D**), and cytoplasmic intensity (**E**) of TDP43 from cells shown in **A**. (**F**) Scatterplot showing TDP43 N/C ratio as a function of FLAG-NLK-WT whole-cell intensity. Data are shown as the mean ± SEM. ***P < 0.0001, unpaired 2-tailed *t* test with Welch’s correction.

To determine whether NLK directly influences TDP43 localization, we transfected HEK293T cells with plasmids encoding FLAG-tagged WT NLK and examined the nuclear and cytoplasmic distribution of TDP43 by immunofluorescence (Figure 1A). As a control, we used a kinase-negative (KN) mutant of NLK, which harbors a point mutation in the kinase domain (K155M) that abrogates its enzymatic activity, as confirmed by loss of autophosphorylation (Supplemental Figure 1A; supplemental material available online with this article; https://doi.org/10.1172/JCI188138DS1) (20). FLAG-NLK-WT and FLAG-NLK-KN were expressed at equivalent levels and displayed a subcellular distribution similar to that of endogenous NLK (Supplemental Figure 1, B–F). Overexpression of NLK-WT, but not NLK-KN, significantly decreased the nuclear/cytoplasmic (N/C) ratio of endogenous TDP43 (Figure 1B). This change was driven by both a reduction in nuclear TDP43 and a concordant increase in cytoplasmic TDP43, consistent with bona fide mislocalization (Figure 1, C–E). To determine whether this effect was dose dependent, we quantified FLAG-NLK-WT levels in single cells and plotted them against TDP43 N/C ratios (Figure 1F). Despite the modest negative correlation between FLAG-NLK-WT expression and TDP43 N/C ratios, we detected prominent TDP43 mislocalization even at the lowest FLAG-NLK-WT levels, suggesting that NLK operates in a largely dose-independent fashion.

**Figure 2:**
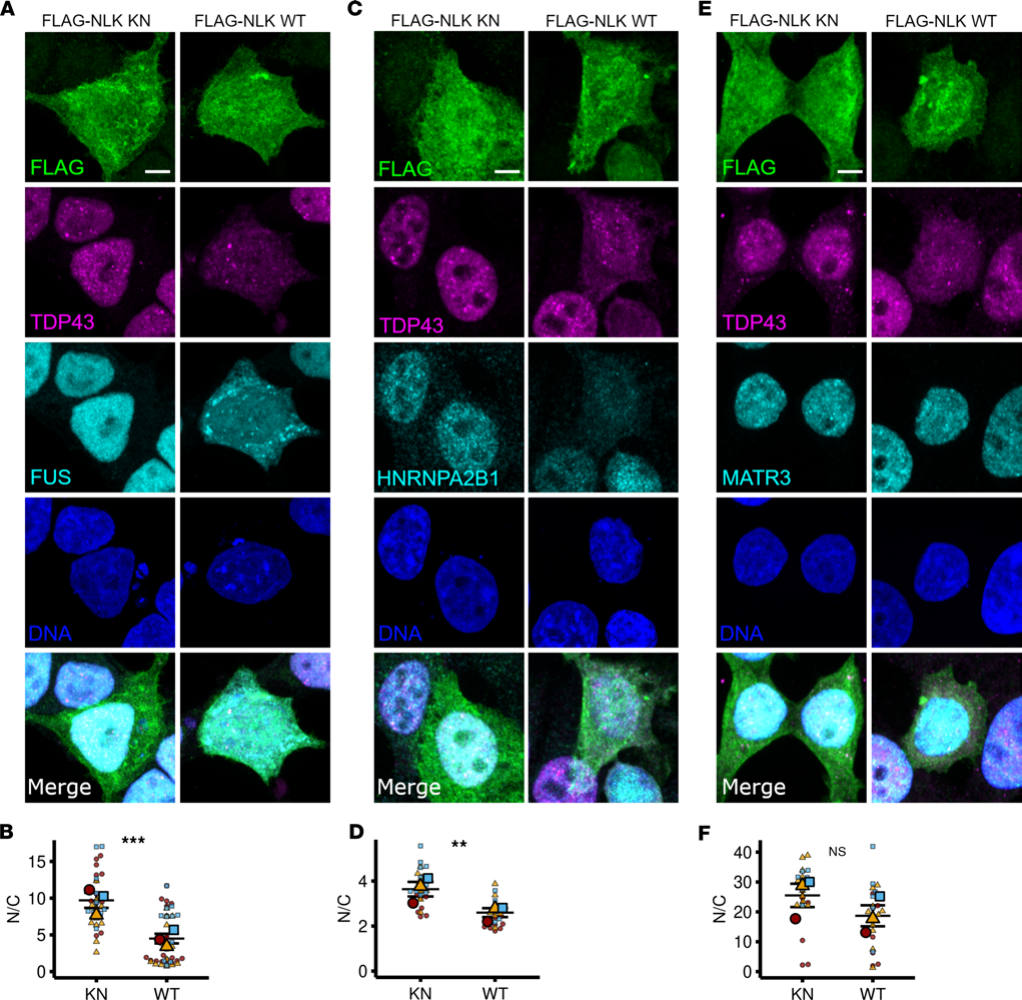
Overexpression of NLK leads to cytoplasmic accumulation of ALS/FTLD–relevant RNA-binding proteins. (**A**–**F**) HEK293T cells were transfected with plasmids encoding either FLAG-NLK-KN or FLAG-NLK-WT, followed by immunofluorescence using antibodies against FLAG (green), TDP43 (magenta), and FUS (**A**), HNRNPA2B1 (**C**), or MATR3 (**E**) (cyan); DNA was stained with Hoechst (blue). Scale bar: 10 μm. (**B**, **D**, and **F**) Superplots of the N/C ratio of FUS (**A**), HNRNPA2B1 (**C**), or MATR3 (**E**) in cells overexpressing KN or WT NLK. Data are shown as the mean ± SEM. **P < 0.01, ***P < 0.0001, unpaired 2-tailed *t* test with Welch’s correction.

We next asked whether NLK solely affects TDP43 localization, or whether it also regulates the distribution of other ALS-associated RNA-binding proteins, including HNRNPA2B1 and Matrin-3 (21, 22). We also examined the localization of FUS, an RNA-binding protein harboring a nonclassical PY-nuclear localization signal (NLS) recognized by importin-β_2_, in contrast with the classical K/R-rich NLSs in TDP43 and Matrin-3 that bind importin-α (23–25). As before, HEK293T cells were transfected with KN or WT NLK, and the subcellular distribution of each protein was determined by immunofluorescence. Compared with NLK KN, NLK WT overexpression significantly reduced the N/C ratio of both FUS and HNRNPA2B1 but had no significant effect on Matrin-3 localization (Figure 2, A–F, and Supplemental Figure 2, H–J). As an additional control, we interrogated the localization of UPF1, a relatively large cytoplasmic protein (26). Immunocytochemistry in HEK293T cells transfected with KN or WT NLK demonstrated that UPF1 localization was unaffected by NLK overexpression (Supplemental Figure 2, K–M). Together, these findings suggest that WT NLK overexpression disrupts localization of multiple RNA-binding proteins harboring classical as well as nonclassical NLSs in a kinase-dependent manner.

### NLK overexpression disrupts nuclear import.

**Figure 3:**
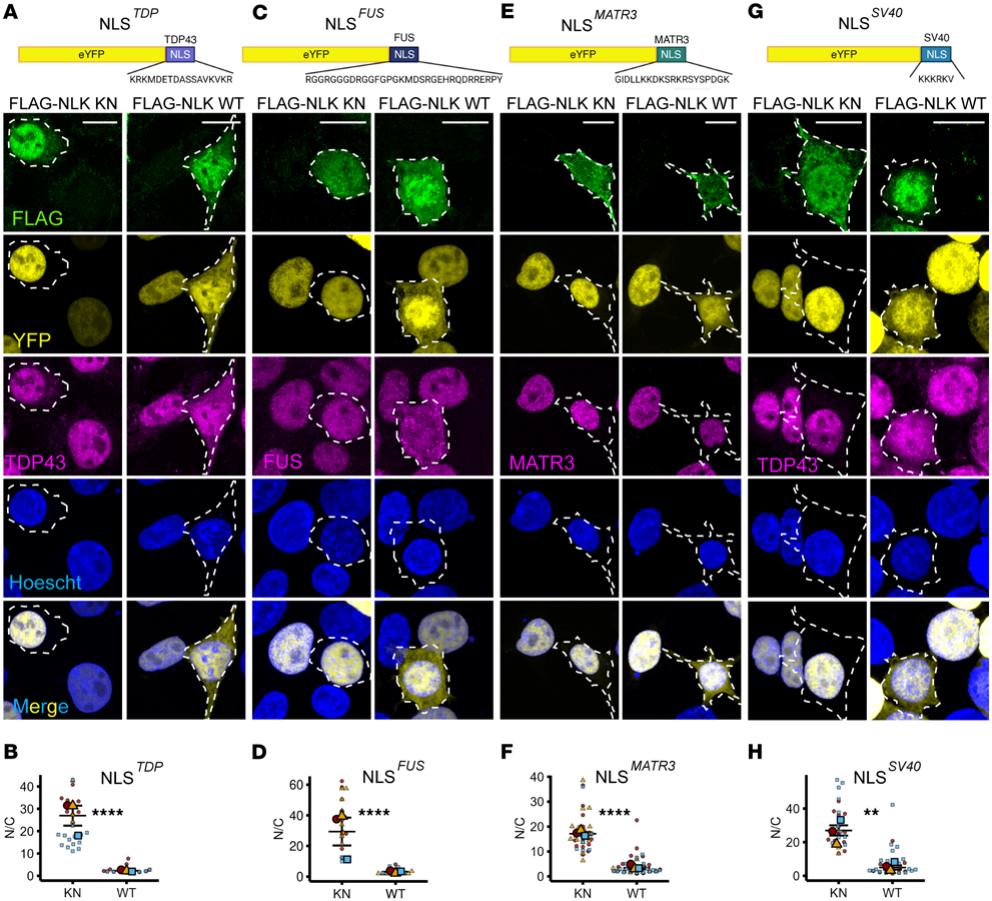
NLK overexpression disrupts NLS-dependent nuclear import. (**A**–**H**) HEK293T cells were cotransfected with plasmids encoding either FLAG-NLK-KN or FLAG-NLK-WT and either of the following: NLS reporter eYFP-NLS^TDP43^ (**A** and **B**), eYFP-NLS^FUS^ (**C** and **D**), eYFP-NLS^MATR3^ (**E** and **F**), or eYFP-NLS^SV40^ (**G** and **H**). Representative images of immunofluorescence are shown in **A**, **C**, **E**, and **G**, using antibodies against FLAG (green) and either TDP43, FUS, or Matrin-3 (magenta); direct fluorescence of reporter fusion protein is shown in yellow. DNA was stained with Hoechst (blue). Scale bar: 10 μm. Superplots of quantification of NLS-reporter localization nuclear/cytoplasmic ratio (N/C) are shown in **B**, **D**, **F**, and **H**. Data are shown as the mean ± SEM. **P < 0.01, ****P < 0.0001, unpaired 2-tailed *t* test with Welch’s correction.

At steady state, nuclear localization of TDP43 and many other RNA-binding proteins is maintained through 2 competing processes: active nuclear import and passive efflux through the nuclear pore (27–29). To directly evaluate nuclear import, we coexpressed NLK together with yello fluorescent protein (YFP) fusion proteins containing NLS sequences from TDP43 (YFP-NLS^TDP^), FUS (YFP-NLS^FUS^), Matrin-3 (YFP-NLS^MATR3^), and SV40 (YFP-NLS^SV40^, a canonical classical NLS), followed by immunofluorescence for TDP43, FUS, or Matrin-3 (Figure 3, A–H). Compared with overexpression of NLK KN, NLK WT significantly increased the N/C ratio of all NLS-fusion proteins (Figure 3, A–H). As we observed for native RNA-binding proteins (Figures 1 and 2), WT NLK affects reporters containing both classical and nonclassical NLS motifs (30). Notably, WT NLK expression drove mislocalization of YFP-NLS^MATR3^ but not endogenous Matrin-3 (Figure 3, E and F), potentially due to the relatively large size of Matrin-3 (95 kDa vs. 27 kDa for YFP-NLS^MATR3^) (27, 28). Collectively, these data indicate that WT NLK overexpression disrupts global nuclear import through its kinase activity.

### NLK-induced TDP43 mislocalization is independent of KPNA2 nuclear accumulation.

**Figure 4:**
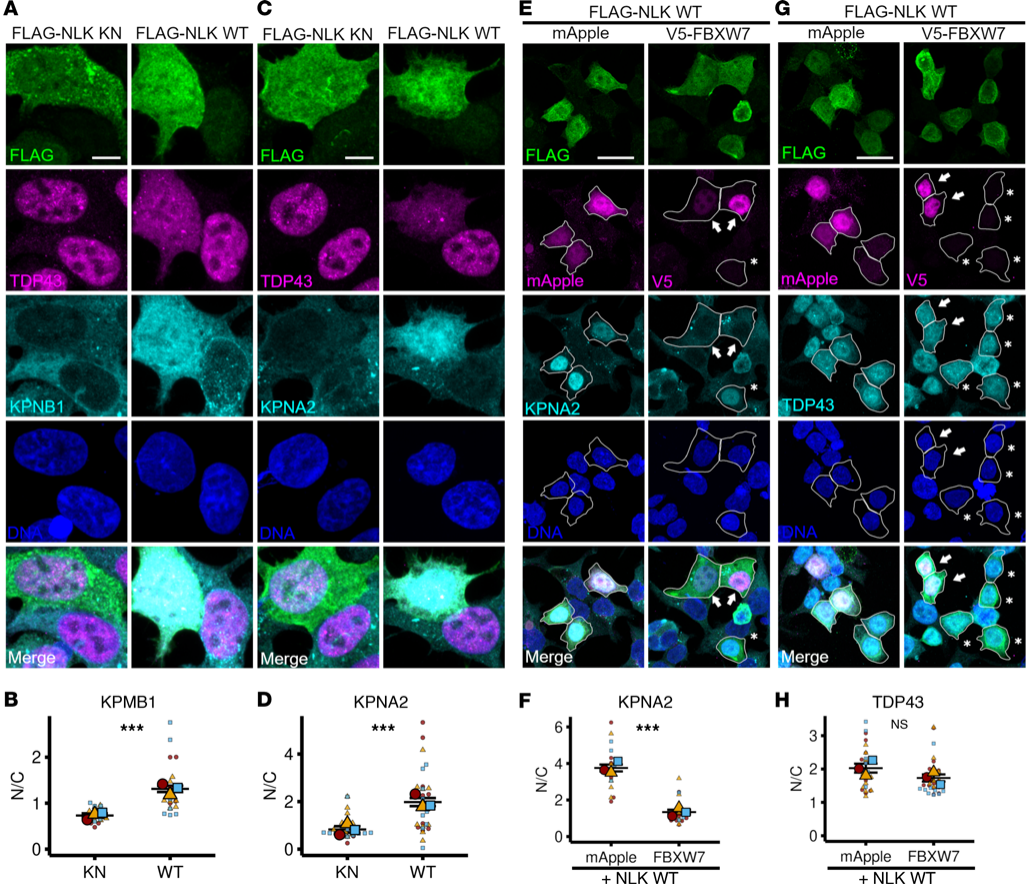
NLK-dependent mislocalization of TDP43 does not depend on nuclear accumulation of KPNA2 and KPNB1. (**A**–**D**) HEK293T cells were transfected with plasmids encoding either FLAG-NLK-KN or FLAG-NLK-WT. Representative images of immunofluorescence are shown in **A** and **C**, using antibodies against FLAG (green), TDP43 (magenta), and KPNB1 (**A**) or KPNA2 (**C**) (cyan); DNA was stained with Hoechst (blue). Scale bar: 5 μm. Superplots of quantification of N/C ratio of the indicated proteins are shown in **B** and **D**. Data are shown as the mean ± SEM. Statistical significance indicated as such in all panels: ***P < 0.001 (unpaired 2-tailed *t* test with Welch’s correction). (**E**–**H**) HEK293T cells were cotransfected with plasmids encoding FLAG-NLK-WT and either mApple (negative control) or V5-FBXW7. Representative images of immunofluorescence are shown in **E** and **G** using antibodies against FLAG (green), V5 (magenta), and KPNA2 (**E**) or TDP43 (**G**) (cyan).Direct fluorescence of mApple is shown in magenta. DNA was stained with Hoechst (blue). In **E** and **G**, arrows indicate cells coexpressing FLAG-NLK WT and V5-FBXW7, while asterisks indicate cells expressing FLAG-NLK WT only. Scale bar: 20 μm. Superplots of N/C ratios of the indicated proteins are shown in **F** and **H**. Data are shown as the mean ± SEM. Statistical significance indicated as such in all panels: ***P < 0.001 (unpaired 2-tailed *t* test with Welch’s correction).

Nuclear import relies on transport receptor proteins, such as KPNA2 and KPNB1, which recognize and bind to NLS-containing proteins such as TDP43 (31). Thus, we examined the subcellular localization of KPNB1 and KPNA2 by immunofluorescence after overexpression of either KN or WT NLK. Overexpression of WT NLK significantly increased the N/C ratio of both KPNA2 and KPNB1, driven by a significant increase in their nuclear fractions without a corresponding decrease in their cytoplasmic abundance (Figure 4, A–D, and Supplemental Figure 3, A and B). Because of the critical importance of these factors for nuclear import, we questioned whether the mislocalization of TDP43 and other RNA-binding proteins may be secondary to the observed nuclear accumulation of KPNA2 or KPNB1. To test this hypothesis, we took advantage of previous data showing that nuclear accumulation of KPNA2 can be reversed by the expression of the E3 ubiquitin ligase FBXW7 (32). HEK293T cells were transfected with plasmids encoding FLAG-tagged NLK WT and either mApple (negative control) or FBXW7, followed by immunofluorescence for KPNA2 (Figure 4E). Compared with mApple, FBXW7-V5 significantly reduced the N/C ratio of KPNA2 in NLK-overexpressing cells (Figure 4F), due primarily to reductions in nuclear KPNA2 (Supplemental Figure 3C). Despite this, FBXW7-V5 coexpression failed to significantly correct TDP43 mislocalization in cells transfected with WT NLK (Figure 4, G and H, and Supplemental Figure 3D). These results indicate that NLK-induced mislocalization of TDP43 does not depend on the nuclear accumulation of KPNA2.

### NLK overexpression promotes mislocalization of Ran, Ran-GAP, and RanBP2.

**Figure 5:**
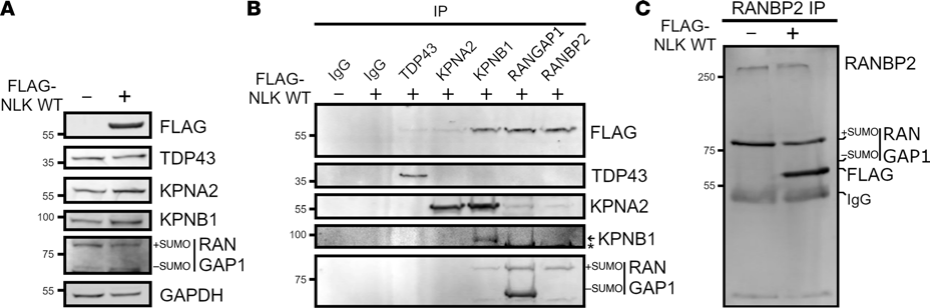
NLK interacts with the RanBP2-RanGAP1 complex. (**A**) Representative Western blots from HEK293T cells transfected with either empty vector (–) or FLAG-NLK-WT (+). Molecular weights in kDa are indicated on the left. (**B**) Western blot analysis after IP of lysates from empty vector (–) or FLAG-NLK-WT–expressing cells using the indicated antibodies. For KPNB1, the arrow indicates the KPNB1-reactive band, and the asterisk (*) indicates SUMO–RanGAP1. (**C**) Higher-resolution Western blot after IP of RanBP2 from empty vector (–) or FLAG-NLK-WT–expressing cells.

**Figure 6:**
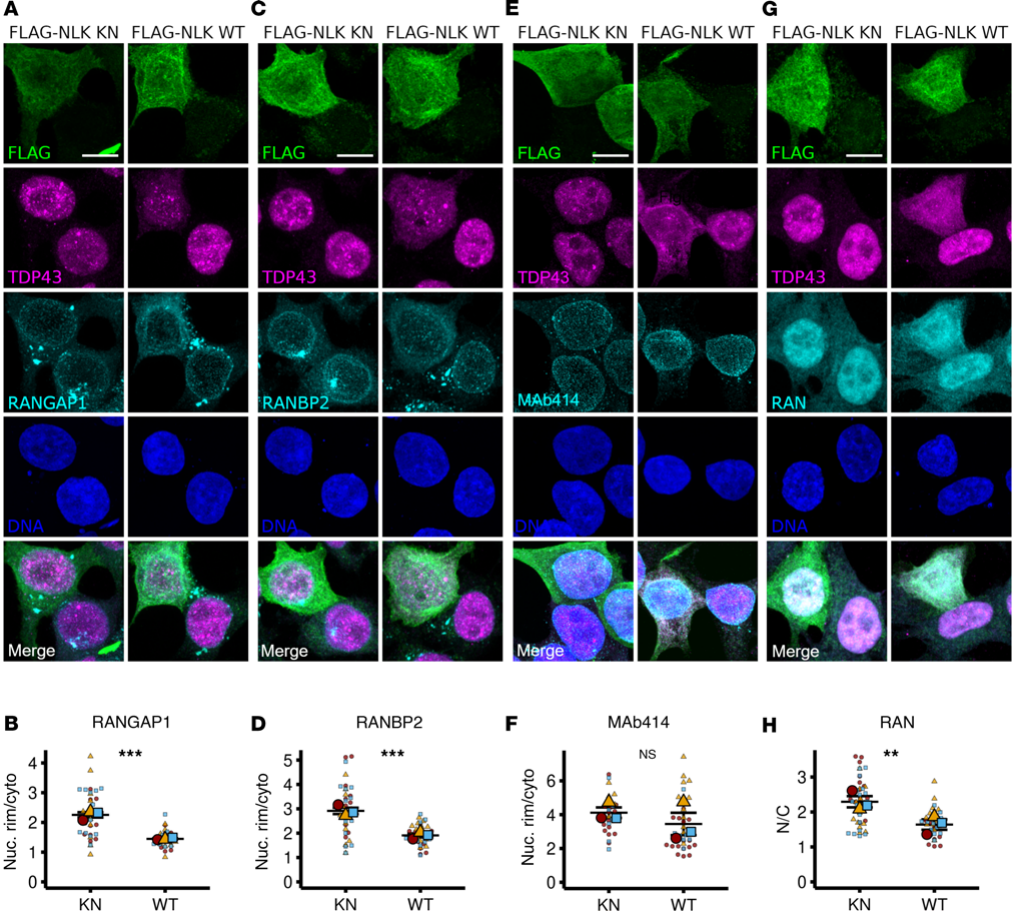
NLK overexpression mislocalizes RanBP2-RanGAP1 and disrupts the Ran gradient. (**A**–**H**) HEK293T cells were transfected with plasmids encoding either FLAG-NLK-KN or FLAG-NLK-WT, followed by immunofluorescence using antibodies against FLAG (green), TDP43 (magenta), and either RanGAP1 (**A**), RanBP2 (**C**), MAb414 (FG-nucleoporins; **E**), or Ran (**G**) (cyan); DNA was stained with Hoechst (blue). Scale bar: 10 μm. (**B**, **D**, **F**, and **H**) Superplots of nuclear rim-to-cytoplasmic ratio (Nuc. Rim/Cyto) of RanGAP1 (**A**), RanBP2 (**C**), and MAb414 (**E**) or nuclear to cytoplasmic ratio (N/C) of Ran (**G**). Data are shown as the mean ± SEM. Statistical significance is indicated as follows in all panels: **P < 0.01, ***P < 0.001. *P* values calculated using unpaired 2-tailed *t* test with Welch’s correction.

Nuclear localization of receptor-bound cargo is mediated by RanGAP1 and RanBP2, nuclear pore–associated factors that regulate the Ran gradient (33). Consistent with a previous screen for kinase-interacting proteins (34), both RanGAP1 and RanBP2 coimmunoprecipitated with FLAG-NLK in HEK293T cells (Figure 5, A–C). NLK overexpression also promoted the accumulation of nonsumoylated RanGAP1 (Figure 5A). RanGAP1 sumoylation is critical for nuclear envelope localization and its interaction with RanBP2 (Figure 5C) (35–37). This prompted us to investigate the impact of NLK overexpression on the subcellular localization of RanGAP1, RanBP2, and ultimately Ran. Compared with KN NLK overexpression, WT NLK significantly disrupted the expected nuclear envelope localization of RanGAP1 and RanBP2 as measured by the ratio of nuclear rim density to cytoplasmic density (Figure 6, A–D). Conversely, overexpression of NLK WT did not significantly affect FG-nucleoporins as detected by MAb414 (Figure 6, E and F). WT NLK overexpression also reduced the N/C ratio of Ran (Figure 6, G and H). As such, NLK-induced disruption of the Ran gradient correlates with disruption of the RanGAP1-RanBP2 complex and impaired nucleocytoplasmic transport.

### NLK drives redistribution of mRNA and disassembles nuclear speckles.

**Figure 7:**
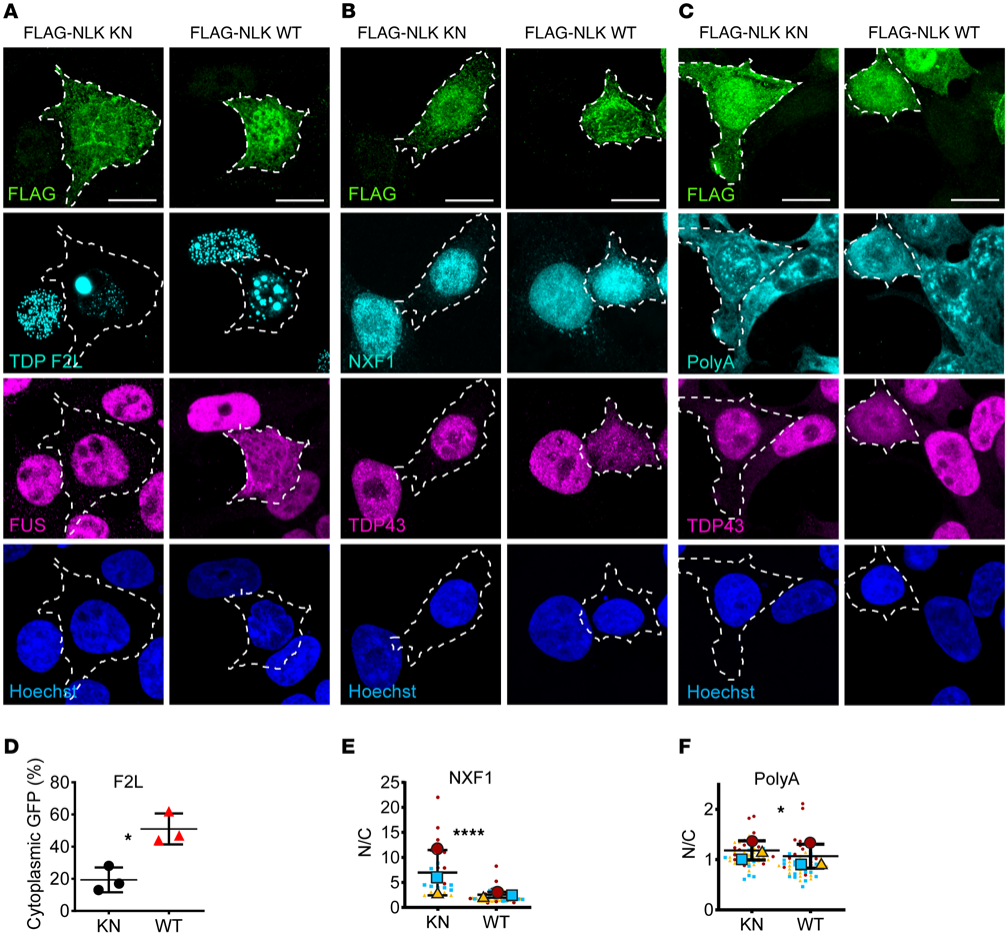
NLK-induced TDP43 mislocalization is RNA independent, but NLK overexpression affects RNA distribution. (**A**) HEK293T cells were cotransfected with plasmids encoding either FLAG-NLK-KN or FLAG-NLK-WT and a GFP-tagged RNA-binding mutant TDP43 (TDP43 F147/9L; F2L), followed by immunofluorescence using antibodies against FLAG (green) and FUS (magenta) and direct visualization of tagged protein (cyan); DNA was stained with Hoechst (blue). Scale bar: 10 μm. (**B**) HEK293T cells were transfected with plasmids encoding either FLAG-NLK-KN or FLAG-NLK-WT, followed by immunofluorescence using antibodies against FLAG (green), TDP43 (magenta), and NXF1 (cyan); DNA was stained with Hoechst (blue). Scale bar: 10 μm. (**C**) HEK293T cells were transfected with plasmids encoding either FLAG-NLK-KN or FLAG-NLK-WT, followed by polyA FISH (cyan) and immunofluorescence for FLAG (green) and TDP43 (magenta); DNA was stained with Hoechst (blue). Scale bar: 10 μm. (**D**–**F**) Quantification of data presented in **A**–**C**. (**D**) Percentage of cells with cytoplasmic GFP TDP F2L signal in cells expressing FLAG-NLK-KN or FLAG-NLK-WT. (**E** and **F**) Superplots of N/C ratio of NXF1 or PolyA FISH in HEK293T cells expressing FLAG-NLK-KN or FLAG-NLK-WT. Data are shown as mean ± SD. *P < 0.05, ****P < 0.0001, unpaired 2-tailed *t* test with Welch’s correction.

The localization of TDP43 and other RNA-binding proteins is heavily influenced not only by their NLS motifs but also by their cognate RNA substrates (38). Based on this, we examined whether TDP43 mislocalization upon WT NLK overexpression is RNA dependent. Initially, we transfected HEK293T cells with an EGFP-tagged variant of TDP43 harboring 2 mutations within RRM1 that abolish RNA binding, TDPF2L-EGFP (F147L/F149L) (39, 40), together with WT or KN NLK (Figure 7A). TDPF2L-EGFP formed phase-separated droplets that were largely restricted to the nucleus in cells coexpressing KN NLK, as in prior studies (41). However, cotransfection with WT NLK resulted in the appearance of cytosolic TDP43F2L-EGFP droplets, suggesting that TDP43 mislocalization upon WT NLK overexpression is independent of RNA binding (Figure 7, A and D).

**Figure 8:**
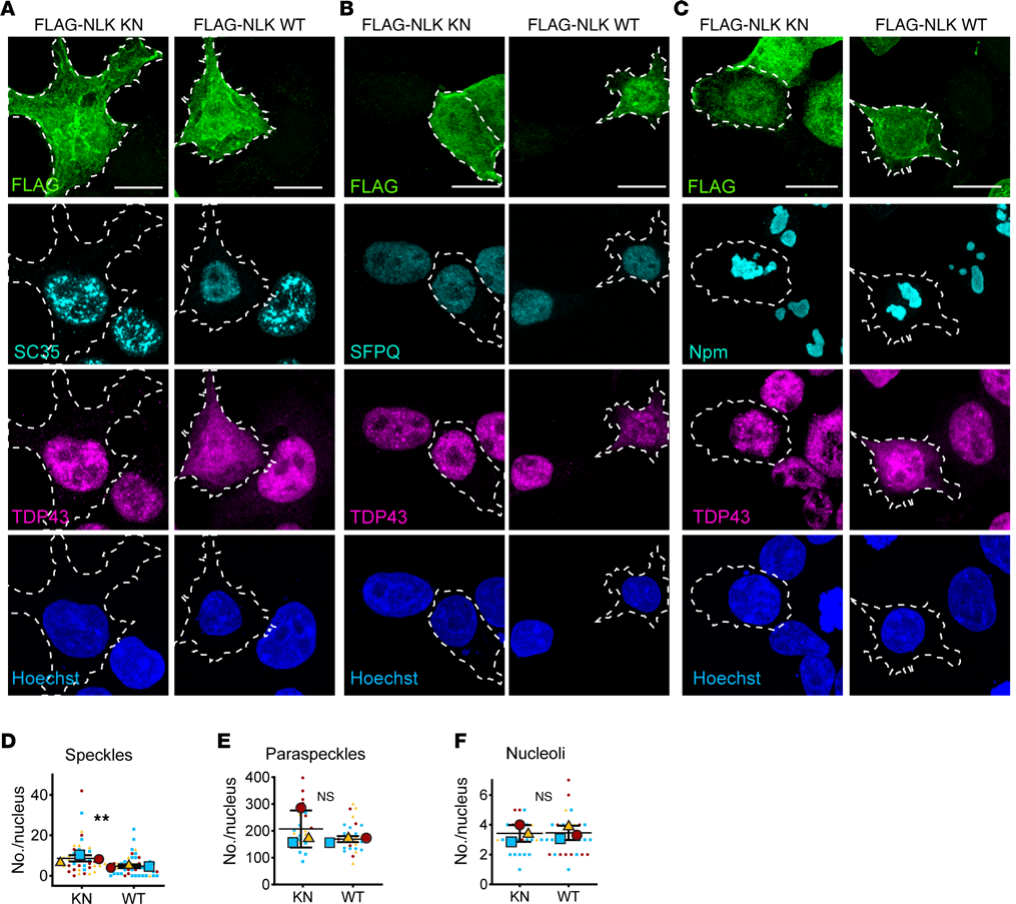
NLK overexpression drives dissolution of nuclear speckles. (**A**–**C**) HEK293T cells were transfected with plasmids encoding either FLAG-NLK-KN or FLAG-NLK-WT. (**A**–**C**) Representative images of immunofluorescence for FLAG (green), TDP43 (magenta), and markers of speckles (SC-35; cyan) (**A**), paraspeckles (SFPQ; cyan) (**B**), or nucleolus (nucleophosmin, Npm; cyan) (**C**). DNA is stained with Hoechst (blue). Scale bar: 10 μm. (**D**–**F**) Superplots of number of speckles (**D**), paraspeckles (**E**), or nucleoli (**F**) in HEK293T cells expressing FLAG-NLK-KN or FLAG-NLK-WT. Data are shown as mean ± SD. **P < 0.01, unpaired 2-tailed *t* test with Welch’s correction.

We also investigated whether WT NLK overexpression affects the distribution of polyadenylated (polyA) mRNA. We first immunostained for NXF1, an mRNA export factor that contains a PY-NLS (23), and saw that in NLK WT–transfected cells, NXF1 accumulated in the cytoplasm (Figure 7, B and E, and Supplemental Figure 4A). Next, we directly assessed mRNA distribution using FISH. While untransfected and KN NLK–expressing cells displayed a punctate pattern of polyA mRNA within the nucleus, WT NLK overexpression resulted in a more diffuse and evenly distributed nuclear signal, with minimal changes in the polyA mRNA N/C ratio (Figure 7, C and F, and Supplemental Figure 4B). Given the enrichment of polyA mRNA within nuclear speckles and the apparent loss of such structures with WT NLK overexpression, we also immunostained WT or KN NLK–transfected cells using the SC35 antibody, which recognizes SRRM2, a core component of nuclear speckles (42). WT NLK–expressing cells, in contrast to untransfected or KN NLK–transfected cells, displayed a dramatic reduction in nuclear speckles (Figure 8, A and D). This effect appeared to be specific for nuclear speckles, as we saw no change in other nuclear membrane–less organelles: paraspeckles (marked by SFPQ; Figure 8, B and E; ref 43) or nucleoli (marked by nucleophosmin; Figure 8, C and F; ref 44) in WT NLK–expressing cells.

### NLK overexpression disrupts nuclear import in mammalian neurons.

**Figure 9:**
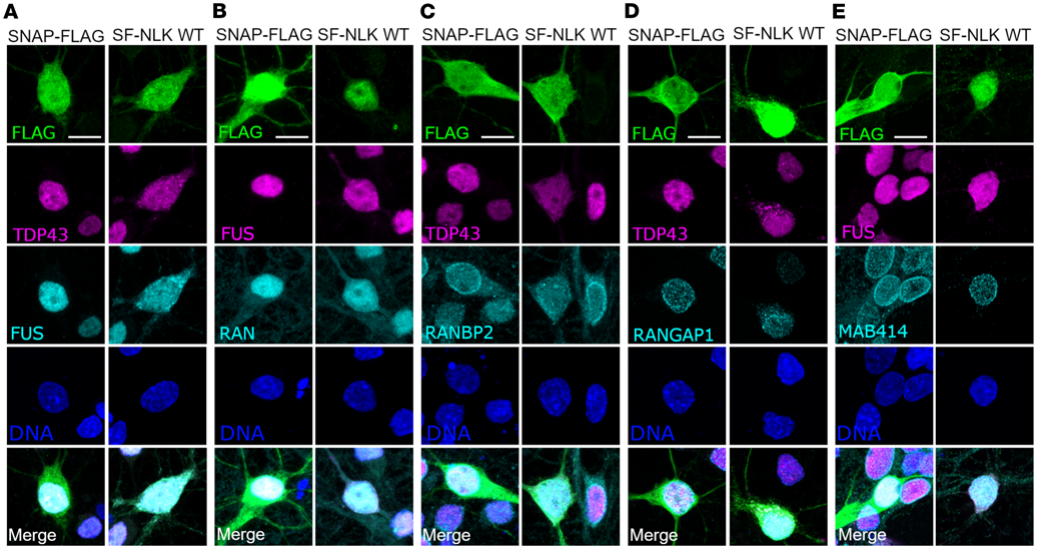
NLK overexpression disrupts nuclear import in primary rat neurons. (**A**–**E**) Primary rodent cortical neurons were transfected with either SNAP-FLAG (SF; negative control) or SNAP-FLAG-NLK (SF-NLK), followed by immunofluorescence using antibodies against FLAG (green) and TDP43, FUS (magenta), Ran, RanBP2, RanGAP1, or MAb414 (cyan); DNA was stained with Hoechst (blue). Scale bar: 10 μm.

To examine the impact of NLK overexpression on RNA-binding proteins and nuclear import factors in neurons, we transfected rodent primary mixed cortical neurons with either SNAP-FLAG (SF; negative control) or SNAP-FLAG-NLK-WT (SF-NLK) before immunostaining for TDP43 and other factors affected by NLK. Consistent with our results in HEK293T cells, expression of SF-NLK but not SF affected the subcellular localization of TDP43, FUS, RanGAP1, RanBP2, and Ran (Figure 9, A–D, and Supplemental Figure 5, A, B, and D). As before, the central channel of the nuclear pore, visualized by MAb414, was unaffected by SF-NLK expression (Figure 9E), while exogenous reporters, such as YFP-NLS^SV40^, were mislocalized by SF-NLK but not SF in transfected neurons (Supplemental Figure 5, F and G).

**Figure 10:**
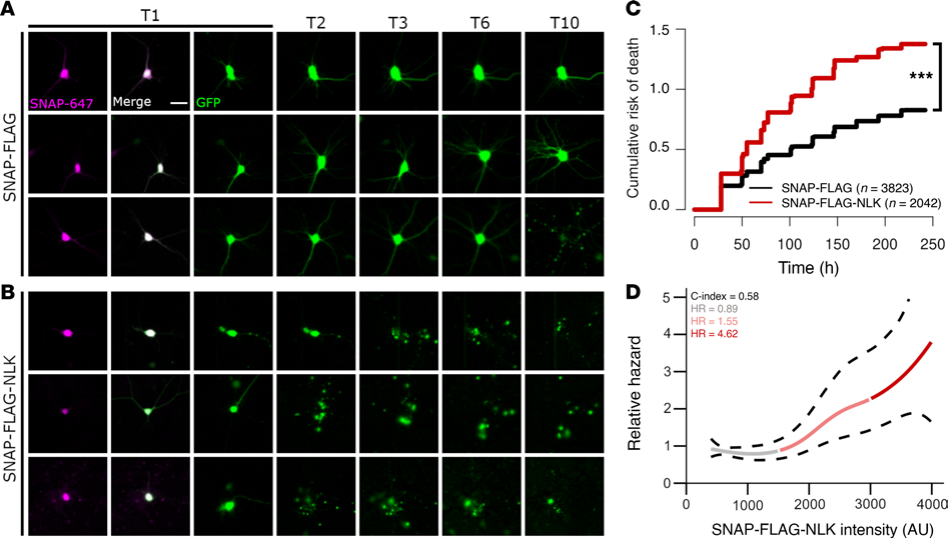
NLK overexpression is toxic in primary rat neurons. (**A** and **B**) Primary rodent cortical neurons were cotransfected with plasmids encoding either SF or SF-NLK and EGFP (survival marker), treated with SNAP-647 dye at T1 to visualize SNAP-positive neurons, and tracked by longitudinal microscopy to determine neuronal fate. Scale bar: 20 μm. (**C**) Cumulative hazard plot showing the relative risk of death in neurons expressing either SF or SF-NLK. HR = 1.616. ***P = 1.494 × 10^–59^. (**D**) Cox proportional hazards model predicting relative hazard based on SNAP-FLAG-NLK expression intensity. Solid lines represent estimated hazard; color gradients reflect expression levels: gray (low), light red (medium), and red (high). Dashed lines represent 95% CI.

Nucleocytoplasmic trafficking is essential for maintaining protein and RNA homeostasis; thus, we predicted that NLK-induced disruption of nucleocytoplasmic transport would lead to substantial toxicity. To assess this, we utilized automated longitudinal microscopy to track hundreds of rodent primary mixed cortical neuron cultures prospectively for 10 days in culture (40, 45–47). Neurons were transfected with SF or SF-NLK in combination with a survival marker (GFP), enabling us to determine the time of cell death (Figure 10, A and B). SF-NLK overexpression significantly increased the cumulative risk of death in transfected neurons compared with SF alone (HR = 1.61, *P* < 0.001, Cox proportional hazards analysis; Figure 10C). Since all data on survival are acquired from individual neurons, and the abundance of fluorescently tagged proteins is directly proportional to the measured signal intensity (48), we investigated the relationship between SNAP-FLAG-NLK intensity and risk of death using a Cox proportional hazards penalized spline model (Figure 10D) (49–51). HRs were calculated for distinct intensity segments derived from the spline model. For low signal intensities, the HR was 0.898 (95% CI: 0.378–2.132), indicating a slight decrease in the relative risk of death compared with the baseline. In the medium intensity range, the HR increased to 1.555 (95% CI: 0.532–4.545), suggesting a potential increase in risk. Notably, for high signal intensities, the HR rose significantly to 4.629 (95% CI: 0.701–30.584), reflecting a substantial elevation in hazard associated with increased NLK expression levels.

To confirm that the TDP43 mislocalization observed with NLK overexpression is specifically associated with NLK-dependent processes, rather than a secondary event observed upon cell death, we also assessed TDP43 localization in primary rodent cortical neurons overexpressing dual leucine zipper kinase (DLK), a key regulator of axon degeneration and neuronal survival (52–54). First, we took advantage of automated longitudinal fluorescence microscopy to verify the toxicity of DLK-GFP in primary neurons. As expected, cells expressing DLK-GFP displayed a significant increase in the risk of death compared with neurons transfected with GFP alone (HR = 2.03, *P* < 2 × 10^–16^, Cox proportional hazards analysis) (Supplemental Figure 5E). Despite this, TDP43 localization was unaffected by DLK-GFP expression (Supplemental Figure 5, C and D). Together, these data indicate that NLK overexpression impairs nucleocytoplasmic transport mechanisms, leading to the mislocalization of pertinent RNA-binding proteins and ultimately neuron death.

### Increased NLK levels correlate with TDP43 pathology in disease models and patients.

**Figure 11:**
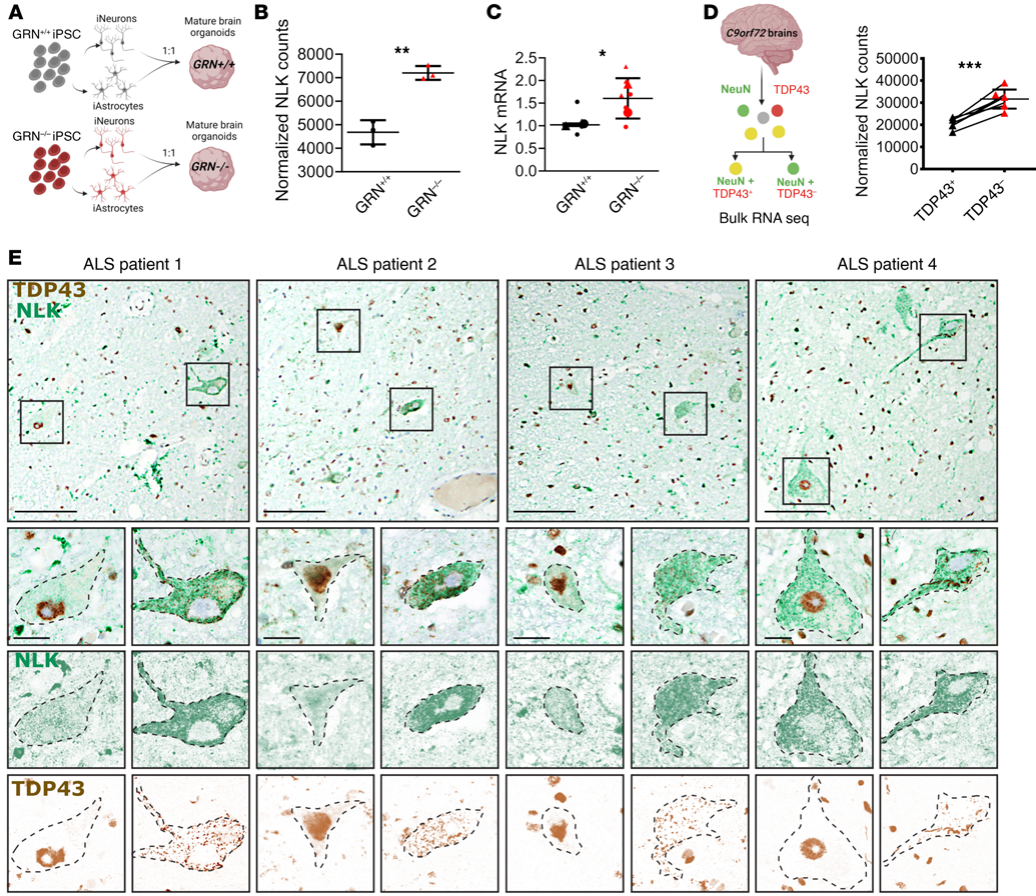
TDP43 pathology is associated with NLK overexpression in human model systems and patient samples. (**A**) Schematic of generation of mbOrgs. (**B**) Normalized NLK counts from RNA-Seq performed on WT or *GRN*^–/–^ mbOrgs. Data are shown as mean ± SD. **P < 0.01, unpaired 2-tailed *t* test with Welch’s correction. (**C**) qRT-PCR analysis of NLK mRNA levels in WT and *GRN*^–/–^ mbOrgs (2 biological replicates, 3 technical replicates per condition). Superplot of NLK expression normalized to GAPDH. Line = mean; error bars = standard deviation. *P < 0.05, unpaired 2-tailed *t* test with Welch’s correction. (**D**) NLK normalized counts from RNA-Seq performed on TDP43-positive and -negative nuclei. Data are shown as mean ± SD. ***P < 0.001, paired 2-tailed *t* test with Welch’s correction. (**E**) Dual IHC for NLK and TDP43, performed on spinal cord tissue from 4 patients with sporadic ALS. Images deconvolved using Fiji. Scale bar for upper panels: 100 μm. Scale bar for lower panels: 20 μm.

Approximately 50% of individuals with frontotemporal lobar degeneration (FTLD) show TDP43 mislocalization as in ALS (2). In addition, up to half of people with ALS demonstrate cognitive impairment reminiscent of FTLD, and approximately one-third of individuals with FTLD show motor neuron disease that is indistinguishable from ALS (2, 55). These observations, as well as shared genetics underlying both ALS and FTLD, testify to the close overlap between ALS and FTLD with TDP43 pathology (FTLD-TDP) (56). Therefore, to determine whether NLK dysregulation may be involved in ALS/FTLD-TDP disease pathogenesis, we initially investigated *NLK* expression in progranulin knockout (*GRN*-KO) mature brain organoids (mbOrgs), an FTLD-TDP model that recapitulates key pathological features of disease, including TDP43 mislocalization, phosphorylated TDP43, and characteristic missplicing of TDP43 substrate RNAs (57). Neurogenin-2 inducible cortical neurons (iNeurons) and mature cortical astrocytes (iAstrocytes) derived from isogenic WT or *GRN*^–/–^ iPSCs were combined in fixed ratios to form mbOrgs (Figure 11A). RNA-Seq revealed significantly elevated normalized counts of *NLK* in *GRN*^–/–^ mbOrgs compared with WT controls (Figure 11B), a finding that was also confirmed by quantitative RT-PCR (qRT-PCR) (Figure 11C).

To further explore the link between NLK changes and TDP43 pathology, we turned to a unique dataset generated by Liu et al. in which neuronal nuclei were sorted from frontal cortices of patients with FTLD-TDP into 2 populations — those with and without nuclear TDP43 — prior to RNA-Seq (58). Reanalysis of these data demonstrated a significant upregulation of *NLK* mRNA in nuclei lacking TDP43 (Figure 11D). Using dual IHC, we verified that neurons from ALS spinal cord sections exhibiting TDP43 pathology (nuclear loss of TDP43 with cytosolic inclusions) exhibited more intense staining for NLK in comparison to unaffected neurons present in the same section (Figure 11E and Supplemental Figure 6C). Dual staining for NLK and TDP43 was also performed in sections from 4 control patients without spinal pathology (Supplemental Figure 6D), showing no clear differences in NLK abundance. These results demonstrate a clear relationship between elevated NLK, at both the mRNA and protein levels, and TDP43 mislocalization in FTLD-TDP and ALS. Together, these data show that NLK is upregulated in human patients and in disease models featuring TDP43 pathology, in accord with our data demonstrating TDP43 mislocalization upon NLK overexpression.

### NLK reduction improves survival in iPSC-derived neuron models of ALS.

**Figure 12:**
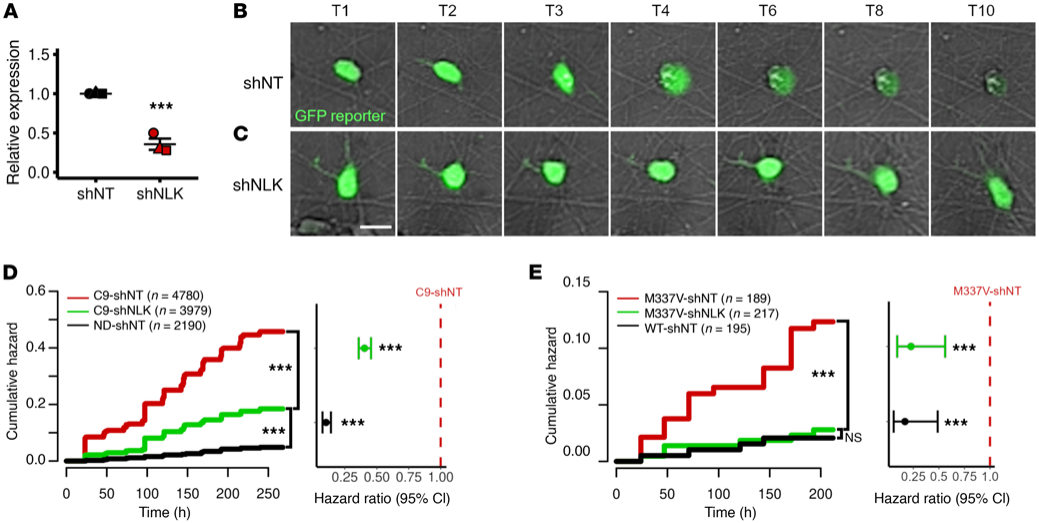
Genetic NLK reduction prevents neurodegeneration in human neuron ALS/FTLD models. (**A**) qRT-PCR for *NLK* mRNA in iPSC-derived neurons transduced with lentivirus encoding either nontargeting shRNA (shNT) or NLK-targeting shRNA (shNLK). Data are shown as the mean ± SEM. ***P < 0.001, unpaired 2-tailed *t* test with Welch’s correction. (**B** and **C**) Isogenic WT and TDP43 M337V iPSC-derived neurons were transduced with lentivirus encoding either shNT or shNLK and tracked by longitudinal microscopy to assess neuronal survival. Scale bar: 20 μm. (**D**) Cumulative hazard plot showing the relative risk of death in ND and C9 neurons expressing either shNT or shNLK. HR = 0.40, ***P < 0.001. (**E**) Cumulative hazard plot showing the relative risk of death in WT and TDP43 M337V neurons expressing either shNT or shNLK. HR = 0.23, ***P = 0.0013.

Given the detrimental effects of NLK overexpression on nucleocytoplasmic transport (Figures 1–9) and neuron survival (Figure 10), and the elevated *NLK* mRNA and protein observed in patients and disease models (Figure 11), we asked whether targeting *NLK* could ameliorate disease phenotypes in ALS/FTLD-TDP models. First, we examined the survival of iPSC-derived neurons carrying *C9ORF72* expansions, the most prevalent mutation underlying familial ALS and FTLD-TDP in Europe and North America (59). Nondisease and *C9ORF72* mutant neurons were transduced with virus encoding nontargeting or *NLK* shRNA, resulting in an approximately 50% reduction in *NLK* mRNA levels compared with nontargeting shRNA (Figure 12A). Individual neurons were then followed by automated microscopy for 10 days, as before (Figure 12, B and C), and differences in survival assessed via Cox proportional hazards analysis. Three separate lines of *C9ORF72* mutant neurons exhibited significantly higher cumulative risks of death compared with unrelated nondisease neurons (Figure 12D and Supplemental Figure 7, A–C). Transduction with *NLK* shRNA significantly reduced the cumulative risk of death in all 3 lines of *C9ORF72* neurons (HR = 0.403, *P* = 7.71 × 10^–50^, Cox proportional hazards analysis).

Given previous evidence linking NLK to lysosomal biogenesis and autophagy (16), we examined lysosomal and autophagy markers (LAMP1, p62, and LC3B) in both *C9ORF72* neurons transduced with *NLK* shRNA and HEK293T cells stably expressing *NLK* shRNA (Supplemental Figure 7, D–F). *NLK* knockdown had no observable effect on any of these markers, however, arguing against direct NLK-dependent regulation of the lysosomal and autophagy pathway in human neurons. To confirm this, we also measured levels of dipeptide repeat (DPR) proteins produced by repeat-associated non-AUG (RAN) translation from the expanded *C9ORF72* locus, since these proteins are autophagy substrates unique to *C9ORF72* mutant cells (60, 61). Levels of 2 DPR proteins, poly-glycine-proline (GP) and poly-glycine-arginine (GR), were unaffected by *NLK* knockdown in *C9ORF72* mutant iNeurons (Supplemental Figure 7, G and H), consistent with the lack of effect of NLK on autophagy or DPR production in these cells.

To examine whether NLK reduction is neuroprotective outside of *C9ORF72* mutations, we utilized an isogenic pair of *TARDBP* mutant iPSC-derived neurons that were created by CRISPR/Cas9 genome engineering (62). In comparison with isogenic controls (WT), *TARDBP* mutant (M337V) neurons transduced with lentivirus expressing nontargeting shRNA exhibited a significantly higher cumulative risk of death (Figure 12E). As with *C9ORF72* mutant neurons, however, transduction with *NLK* shRNA-expressing virus significantly extended the survival of M337V neurons (HR = 0.227, *P* = 0.0013, Cox proportional hazards analysis). Collectively, these data imply that NLK overexpression drives toxicity in association with TDP43 mislocalization, and NLK reduction promotes neuronal survival in models of ALS and FTLD-TDP.

## Discussion

Our findings indicate that NLK overexpression disrupts the nuclear import and localization of TDP43 and related RNA-binding proteins, including FUS and HNRNPA2B1, in a kinase-dependent manner. These effects correlate with the mislocalization of the RanBP2-RanGAP1 complex and collapse of the Ran gradient, both of which are crucial for functional nucleocytoplasmic transport. As expected based on these observations, NLK dose-dependently increased the risk of death in primary rodent neurons. Notably, we uncovered elevated NLK expression at the RNA and protein levels selectively in neurons with TDP43 pathology both at the RNA level (in FTLD-TDP frontal cortex) and at the protein level (in ALS spinal cord). NLK was also upregulated in a brain organoid model of FTLD-TDP, and reducing NLK in human iPSC-derived neurons carrying disease-associated mutations prolonged survival. These results suggest that NLK may contribute to the pathology of ALS/FTLD-TDP by impairing nucleocytoplasmic transport and promoting neurotoxicity. Targeting NLK protein levels or kinase activity could thus represent a potentially novel therapeutic approach for ALS, FTLD-TDP, and other TDP43 proteinopathies.

Disruption of the Ran gradient, mislocalization of nuclear pore components, and disturbance of nuclear pore architecture — all of which we observed upon NLK overexpression — have likewise been described in ALS/FTLD-TDP samples and disease models (10–12, 63). There is no predicted NLK phosphorylation site within TDP43 itself; rather, we suspect that NLK may interfere with the nucleocytoplasmic transport of several RNA-binding proteins and other factors by acting on integral components of the nuclear pore. We and others noted a direct interaction between NLK and the RanGAP1-RanBP2 complex (ref 34; see Figure 5), which is critical for maintaining the Ran gradient and nuclear import/export (64). NLK overexpression prevented RanGAP1 sumoylation (Figure 5A) and reduced the amount of RanGAP1 in complex with RanBP2 (Figure 5C), consistent with previous observations that sumoylated RanGAP1 is unable to bind RanBP2 (Figure 5C) or localize to the nuclear envelope (Figure 6, A and B) (35–37). One possibility is that NLK directly phosphorylates RanGAP1, inhibiting its GAP activity as well as its sumoylation, thereby disrupting its interaction with RanBP2 at the nuclear pore. Alternatively, NLK may negatively regulate Ubc9, a SUMO E3 ligase required for recruitment of RanGAP1 to the RanBP2 complex (36). None of these possibilities are mutually exclusive, however, as NLK may affect nucleocytoplasmic transport through multiple overlapping mechanisms.

At baseline, NLK is highly expressed in the CNS, and its expression is upregulated by oxidative and osmotic stress (65, 66), conditions associated with the cytosolic accumulation of TDP43 and other, predominantly nuclear, RNA-binding proteins. NLK interacts with several proteins associated with neurodegenerative diseases, including poly-Q expanded androgen receptor in spinobulbar muscular atrophy and ataxin-1 in spinocerebellar ataxia 1 (17, 18); accumulations of these proteins may also contribute to changes in NLK expression or activity. Although previous studies have failed to detect NLK upregulation in disease, in most cases these investigations are limited to evaluation of NLK levels (RNA or protein) in bulk tissue. In contrast, our work revealed NLK upregulation solely within affected neurons displaying TDP43 pathology, in association with evidence of disrupted nucleocytoplasmic trafficking in the same cells, suggesting that NLK expression changes are restricted to cells with TDP43 redistribution. At least 2 hypotheses, which are not mutually exclusive, could account for increased levels and activity of NLK in disease. First, *NLK* pre-mRNA contains several TDP43-binding sites, suggesting that NLK may be directly regulated by TDP43. Alternatively, the upregulation of *NLK* mRNA could occur at the transcriptional level. The promoter regions of the *NLK* gene contain stress-related and neuron-specific sequences, suggesting that transcriptional dysregulation or adaptation within these transcription factor families may drive chronic upregulation of NLK, contributing to its toxicity. Outside of changes in expression, NLK may also be inappropriately activated in disease via diverse stimuli. Pro-inflammatory factors such as TGF-β, IL-6, and Wnt all activate NLK through MAPK-dependent signaling (67). Notably, TGF-β is upregulated in ALS (68), and aberrant Wnt signaling is associated with TDP43 mislocalization in cellular and animal models of disease (69).

NLK is an essential kinase — homozygous *Nlk* deletion results in death in utero or postnatally, depending on the mouse strain (70, 71). However, conditional *Nlk* KO in adult animals is well tolerated (72), and *Nlk* haploinsufficiency (*Nlk*^+/–^) is protective in several neurodegenerative disease models, including TDP43-overexpressing mice (16–18). Similarly, partial *NLK* knockdown in our investigations was associated with extended survival in *C9ORF72* and *TARDBP* mutant human iPSC-derived neurons and was safe in control neurons. These data indicate that even modest reductions in *NLK* may be sufficient for mitigating neurodegeneration in ALS and FTLD-TDP.

Although previous interaction studies highlighted several potential partners for NLK, its substrates in neurons and phosphorylation sites within these substrates remain largely unexplored. RanBP2 and RanGAP1 are 2 potential targets for NLK with clear connections to nucleocytoplasmic transport, but NLK is also likely to act on distinct substrates and disease-related pathways, including the integrated stress response and autophagy (16). One advantage of therapeutic strategies that act on upstream signaling factors such as NLK is the capacity to influence several neuroprotective mechanisms simultaneously. Nevertheless, not all downstream events are likely to be beneficial (19), emphasizing the need for further investigations into NLK targets and the impact of NLK-mediated phosphorylation on their function and contribution to disease.

## Methods

### Sex as a biological variable.

Our study examined tissue from both male and female patients, and similar findings are reported for both sexes.

### HEK293T cell culture and transfection.

HEK293T cells from ATCC (https://www.atcc.org/products/crl-3216) were cultured in DMEM (Gibco, 11995065) supplemented with 10% FBS (Gibco, ILT10082147) at 37°C in 5% CO_2_. Cells were transfected with Lipofectamine 2000 (Invitrogen, 11668027) according to the manufacturer’s instructions. For experiments with YFP-NLS reporters, the lipofectamine solution was divided to ensure there would be cells cotransfected with NLK and reporter as well as cells transfected with the reporter alone. Of the lipofectamine solution, 75% contained 2 plasmids (NLK and reporter), and the remaining 25% contained reporter alone. The lipofectamine solution was then combined and briefly mixed before adding directly to cells. After 24 hours, transfected cells were split in media containing poly-d-lysine (1:500, MilliporeSigma, A-008-E) onto coverslips coated with laminin (1:100, MilliporeSigma, L2020-1MG). Cells were cultured an additional 24 hours, and then immunocytochemistry was performed as detailed below.

### Primary neuron cell culture and transfection.

Cortices from E19–E20 Long-Evans rat embryos or E17–E18 C57BL/6 mouse embryos were dissected and disassociated, and primary neurons were plated at a density of 6 × 10^5^ cells/mL in 96-well plates or in a 24-well plate on coverslips. At in vitro day 4, neurons were transfected using Lipofectamine 2000 as previously described, using equivalent amounts of DNA as used in longitudinal survival studies (62, 73). For experiments with the YFP-NLS reporter, the transfection mix was split as described above for HEK293T cells. After transfection, cells were placed in Neurobasal Complete Media (Gibco, 21103-049), 1× B27 supplement (Gibco, 17504-044), and 1× Glutamax, 100 U/mL penicillin/streptomycin). Cells were fixed for immunocytochemistry 48 hours after transfection with SF or SF-NLK and 18 hours after transfection with DLK-GFP.

### iPSC maintenance and differentiation.

To create neural progenitors, iPSCs were dissociated using Accutase, counted, and plated at 32,000 cells/mL in E8 media with ROCK inhibitor. Differentiation was then induced with doxycycline for 48 hours (2 μg/mL; Sigma-Aldrich, D3447). Differentiating neural progenitors were dissociated using Accutase after 2 days of doxycycline induction and frozen for future experiments.

### Differentiating neural progenitors.

Previously frozen neural progenitors were thawed in E8 media with ROCK inhibitor and incubated for 24 hours. Subsequently, the media was changed to N2 media containing 1× N2 supplement (Gibco, 17502-048), 1× nonessential amino acid (NEAA) supplement (Gibco, 11140-050), 10 ng/mL brain-derived neurotropic factor (BDNF) (PeproTech, 450-02), 10 ng/mL NT3 (PeproTech, 450-03), 0.2 μg/mL laminin (Sigma-Aldrich, L2020), and 2 μg/mL doxycycline (Sigma-Aldrich, D3447). On day 2, the media were replaced with transition media (1:1 full E8 media and DMEM/F12; Gibco, 11320-033). On day 3, the media were switched to B27 media, prepared with 1× B27 supplement (Gibco, 17504-044), 1× Glutamax supplement (Gibco, 35050-061), 10 ng/mL BDNF, 10 ng/mL NT3, 0.2 μg/mL laminin, 1× CultureOne (Gibco, A33202-01), and Neurobasal-A (Gibco, 12349-015). On day 6, cells were transduced with the virus (University of Michigan Vector Core) and maintained in the same media for the remainder of the experiment.

### iPSC cell lines.

The cell lines 1021 and 793 (controls) and 883 and 321 (C9ORF) were generated and characterized as previously described (7). The CS52i and corrected cell lines were obtained from Cedars Sinai. See “iPSC lines” in Supplemental Methods for additional details.

### mbOrg generation.

Isogenic human iPSC lines WTC11 (*GRN*^+/+^ and *GRN*^−/−^) were generated by Bruce R. Conklin from Gladstone Institute of Cardiovascular Disease, San Francisco, California, USA, as previously described (74). iPSCs were cultured and maintained in StemMACS iPS-Brew XF (Miltenyi Biotec, 130-104-368) media on 6-well cell culture plates (GenClone, 25-105MP) coated with Vitronectin (Gibco, A14700) in Dulbecco’s PBS (DPBS). iPSCs were dissociated and passaged using EDTA (Invitrogen, AM9260G) in DPBS.

iAstrocytes and iNeurons were differentiated separately, plated in a 1:1 ratio, and aged for 4 weeks as previously described (46). For iNeuron differentiation, iPSCs were transduced with NGN2-expressing lentivirus constructs, and a previously published protocol was followed for differentiation (61). Briefly, iPSCs were expanded, dissociated, and replated on Matrigel-coated plates. Cells were grown in specialized iNeuron induction media (DMEM-F12 plus Glutamax; Gibco, 10565-018), N-2 supplement (Gibco, 17502-048), and MEM-NEAA (Gibco, 11140-050) containing doxycycline (Sigma-Aldrich, D3072) for 72 hours, with media changed every 24 hours. Cells were then dissociated using Accutase and plated together with iAstrocytes. Cortical-like iAstrocytes were generated as previously described (62). Human iPSCs (*GRN*^+/+^ and *GRN*^–/–^) were grown on vitronectin-coated tissue culture plates using StemMACS iPS-Brew XF (Miltenyi Biotec, 130-104-368) media. On day 0 of differentiation, iPSCs were dissociated into small aggregates and transferred in neurosphere induction medium (NIM) (DMEM-F12/Neurobasal-A at 1:1) plus SMAD inhibitors SB431542 and DMH1. On day 7, spheroids were transferred to Matrigel-coated tissue culture plates with NIM. On day 14, rosette clusters were mechanically removed and transferred to tissue culture flasks with NIM plus basic FGF (PeproTech, 100-18B). Media were changed every 72 hours. On day 20, spheroids were triturated into a single-cell suspension and transferred to a new untreated cell culture flask with astrocyte media (DMEM-F12, Gibco, 10565-018; N2 supplement, Gibco, 17502-048; B27-vitamin A supplement, Gibco, 12587-010; heparin, STEMCELL Technologies, 07980; and Y27632, Tocris Bioscience, 1254). From day 28 to 180, spheroid aggregates were maintained in suspension with astrocyte media plus EGF and FGFb (PeproTech, 100-15 and 100-18B) with media changes every 4–5 days. Spheroid aggregates were triturated every 7–10 days and transferred to new untreated tissue culture flasks. Spheroids were triturated every 7–10 days and transferred to new tissue culture flasks. To generate mbOrgs, iNeurons and iAstrocytes were plated together at a 1:1 ratio on Matrigel-coated 24-well plates at a collective density of approximately 1 × 10^6^ cells/well. Cells were maintained in BrainPhys Complete medium with a 50% medium change every 72 hours. mbOrgs were created on 384-microwell plates as described previously (46), resulting in mbOrgs that were highly uniform in size (~500 µm) after brief spin-down and 1–2 days of subsequent culture. These 3D cocultures were aged for 4 weeks.

### Longitudinal microscopy.

At in vitro day 4, neurons were transfected with 100 ng of a control fluorescent plasmid to mark cell bodies and 100 ng of NLK or 50 ng of DLK using Lipofectamine 2000 as previously described (50, 62, 75). Neurons were imaged as described previously (45, 50, 62, 73) using a Nikon Eclipse Ti inverted microscope with PerfectFocus3a 20× objective lens and either an Andor iXon3 897 EMCCD camera or Andor Zyla 4.2 (+) sCMOS camera. A Lambda CL Xenon lamp (Sutter) with 5 mm liquid light guide (Sutter) was used to illuminate samples, and custom scripts written in BeanShell for use in Micro-manager (open source) controlled all stage movements, shutters, and filters. For automated analyses of primary neuron survival, custom ImageJ (NIH) and Fiji macros, along with Python scripts, were used to identify neurons and draw cellular regions of interest (ROIs) based upon size, morphology, and fluorescence intensity. Custom Python scripts were used to track ROIs over time, and cell death marked a set of criteria that included rounding of the soma, loss of fluorescence, and degeneration of neuritic processes (45, 46). For iNeuron survival, bright-field and fluorescent images for survival experiments started on day 14 and continued for 10 days. For manual analysis of iNeuron survival, image time series were processed by flat-field correction and image registration, followed by programmatic deidentification for blinded analysis. Time-series images were uploaded to a browser-based server where a trained user manually counted survival using the point-tracking mode. GFP-positive cells were identified at time point 1, and cell fate was tracked in bright-field images, where neuron death (uncensored event) was recorded at the appropriate time point. Living neurons at the final time point were considered right-censored. A Cox proportional hazards model was applied to the datasets, stratifying by biological replicates, and the corresponding hazard plots were generated in R.

### Immunocytochemistry.

Cells were fixed with 4% paraformaldehyde (Sigma-Aldrich, P6148) for 10 minutes, rinsed with PBS, and permeabilized with 0.1% Triton X-100 (Bio-Rad, 161-0407) for 10 minutes. Cells were then blocked in 3% BSA (Thermo Fisher Scientific, BP9703-100) in PBS at room temperature for 1 hour before incubation overnight at 4°C in primary antibody diluted in 3% BSA. (See “Antibodies” in Supplemental Methods for additional details.) Cells were then washed 3 times in PBS and incubated at room temperature with secondary antibodies diluted 1:250 in 3% BSA for 1 hour. After 3 washes in PBS containing 1:10,000 Hoechst 33258 dye (Invitrogen, H3569), cells were mounted on Superfrost Plus microscope slides (Thermo Fisher Scientific, 1255015) with ProLong Gold Antifade mounting reagent (Thermo Fisher Scientific, P10144) and imaged as described below.

### Western blotting.

HEK293T cells were collected in PBS and pelleted at 10,000*g* for 5 minutes at 4°C and lysed in RIPA Buffer (Thermo Fisher Scientific, 29900) plus protease inhibitor cocktail (cOmplete, Mini, EDTA-free protease inhibitor cocktail, MilliporeSigma, 118361700021) on ice for 30 minutes. Lysates were centrifuged at 21,000*g* for 15 minutes at 4°C, supernatants were transferred to fresh tubes, and equal protein amounts of each sample across conditions were diluted in 10× sample buffer (10% SDS, 20% glycerol, 0.0025% bromophenol blue, 100 mM EDTA, 1 M DTT, 20 mM Tris, pH 8.0) and heated at 55°C for 3 minutes. Samples were run on SDS-PAGE gels, transferred to PVDF membrane (MilliporeSigma, IPFL0010), blocked with 3% BSA in 0.2% Tween 20 (MilliporeSigma, P9614) in Tris-buffered saline (TBST) for 20 minutes, and blotted overnight at 4°C with primary antibody in 3% BSA in TBST (See “Antibodies” in Supplemental Methods for concentrations). The following day, blots were washed 3 times in TBST and incubated at room temperature for 1 hour in secondary antibodies diluted 1:8,000 in 3% BSA in TBST. Blots were then washed with TBST 3 times and imaged using an Odyssey CLx System (LI-COR).

### Phos-tag acrylamide SDS-PAGE.

HEK293T cells were transfected with the plasmids indicated in figure legend. After 48 hours, cells were lysed in RIPA buffer containing protease and phosphatase inhibitor cocktails for 20 minutes at 4°C and centrifuged at 20,817*g* for 15 minutes. The protein concentration of the supernatant was measured using a Bio-Rad protein assay (catalog 5000006). Lysates were then supplemented with 1 mM MnCl_2_ and run on a Mn2+ Phos-Tag polyacrylamide gel (100 μM Phostag, 10% acrylamide), followed by Western blotting as previously described.

### Immunoprecipitation.

HEK293T cells were transfected with the plasmids indicated in figure legend. After 48 hours, cells were lysed in 0.8 mL of lysis buffer (PBS containing 1 mM CaCl_2_, 0.5 mM MgCl_2_, 1% Triton X-100, protease inhibitor cocktail, and 1 μM pepstatin A) and centrifuged at 20,817*g* for 15 minutes. The protein concentration of the supernatant was measured using a Bio-Rad protein assay (catalog 5000006). The cell lysates (2 mg) were immunoprecipitated with 1 μg of the indicated antibodies overnight at 4°C. The antibodies were precipitated with 30 μL of protein G beads (Roche Diagnostics) at 4°C for 2 hours. The beads were washed 5 times with lysis buffer, and the immuno-isolated materials were eluted by heating at 55°C for 3 minutes in nonreducing SDS sample buffer. Proteins were resolved by SDS-PAGE followed by Western blotting as previously described.

### FISH.

Cells were fixed with 4% paraformaldehyde for 10 minutes, rinsed with PBS, and permeabilized with 0.1% Triton X-100 (Bio-Rad, 161-0407) for 10 minutes. Cells were then washed with 2× SSC (2× 5 minutes) and incubated in hybridization buffer for 2 hours at 37° (8.75% dextran, 1.75× SSC, 17.5% formamide, 0.5 μg/μL yeast tRNA, 10 mmol ribonucleoside vanadyl complex, 0.1% BSA, 0.002 μg/μL Cy3-oligo [dT] 30 probe). Cells were then washed with successive SSC rinses (4× SSC 15 minutes, 2× SSC 15 minutes, 2× SSC 15 minutes). Next, immunocytochemistry was performed as above, beginning with blocking in 3% BSA. All solutions prior to hybridization were diethyl pyrocarbonate treated.

### IHC.

Duplex IHC was performed on a Ventana Discovery Ultra stainer. Slides were dewaxed, rehydrated, and subjected to heat-induced epitope retrieval onboard the stainer. Slides were then subjected to sequential incubation with NLK (rabbit polyclonal antibody, Abcam, Ab26050, 1:250, 32 minutes) and polymer goat anti-rabbit IgG conjugated to HRP (Ventana 760-4311) and developed with Discovery Green chromogen (Ventana). After an additional round of heat-induced epitope retrieval to remove the NLK primary antibody-secondary antibody complex, the slides were stained with TDP43 (rabbit polyclonal antibody, Proteintech, 10782-2-AP, 1:2,000, 20 minutes) and polymer goat anti-rabbit IgG conjugated to HRP and developed with Discovery Brown chromogen (Ventana 760-271). Slides were then counterstained with hematoxylin and coverslipped.

### RNA isolation for bulk RNA-Seq and RT-PCR.

RNA extraction was performed using the TRIzol/phenol-chloroform method (Sigma-Aldrich, T9424) as previously described (62) and according to manufacturer specifications. Each sample contained approximately 60 mbOrgs, totaling approximately 3 × 10^6^ of cells per sample. The extracted RNA was used as a template for the synthesis of cDNA through reverse transcription, using iScript cDNA Synthesis kit (Bio-Rad,1708891) according to the manufacturer’s protocol.

### qRT-PCR.

cDNA samples were treated for genomic DNA contamination using DNA-free DNA Removal kit (Invitrogen, AM1906) per the manufacturer’s instructions. The cDNA was then diluted to a concentration of 5 ng/μL, and 4 μL of each sample (total of 20 ng) were aliquoted in a MicroAmp Optical 96-well reaction plate (Thermo Fisher Scientific, N8010560) in technical duplicates. Samples were processed using 2× SYBR Green qPCR Master Mix assay, and qRT-PCR was run on QuantStudio 6 Real-Time PCR system following the manufacturer’s instructions. Data analysis was carried out applying the Pfaffl mathematical model for relative transcript quantification (76) using GAPDH as a housekeeping gene.

### RNA-Seq.

RNA-Seq and analysis of RNA integrity were analyzed on an Agilent 2100 Bioanalyzer using RNA 6000 Nano Kit (Agilent, 5067-1511). Only samples with an RNA integrity number of 9.4 or greater were used to perform bulk RNA-Seq. Nugen Universal Plus (Tecan) was used as a library kit, and libraries were sequenced on a SP300 flow cell of the Illumina NovaSeq 6000 machine with a paired-end 150 bp sequencing strategy (average depth 90 million reads/sample) at UCSF Genomics Core Facility. Genome was aligned to Ensembl Human.GRCh38.103. Kallisto 0.46.01 was used to generate transcript abundance files for each sample. Transcript count files for each sample were generated using tximport, and transcript differential analysis was performed using DESeq2 v1.24.0. A total of 6 samples were spread across 2 conditions.

### Confocal microscopy.

Confocal images were taken on a Nikon AXR NSPARC confocal system with a 60× NA1.42 Oil/DIS Plan-Apochromat Lambda D objective with a working distance of 1.5 mm and a 40× CFI Apochromat LWD Lambda S objective with a working distance of 0.30 mm.

### Light microscopy.

Whole-slide images were generated by the University of Michigan Digital Pathology group within the Department of Pathology using an Aperio AT2 scanner (Leica Biosystems) equipped with a 20× NA 0.75 Plan Apochromat objective; 40× scanning was achieved using a 2× optical magnification changer. Resolution was 0.25 μm/pixel for 40× scans. Focus during the scan was maintained using a triangulated focus map built from individual focus points determined in a separate step before scanning was started. Proprietary software was used for image processing during the acquisition.

High-quality images for figures were acquired on an Olympus BX51 light microscope equipped with a UPlanSApo100× oil objective with a numerical aperture of 1.40 and a working distance of 0.12 mm. Image deconvolution was performed using Fiji.

### Plasmids.

FLAG-NLK-WT and FLAG-NLK-KN were a gift from Tohru Ishitani Ishitani (Unit on Nervous Development Systems, Nagoya, Japan) (77). SNAP-FLAG and SNAP-FLAG-NLK were synthesized and subcloned into FUGW. All YFP reporter plasmids were derived from EYFP2-SV40NLS-NES, a gift from Yuh Min Chook (Department of Pharmacology, UT Southwestern Medical Center at Dallas, Dallas, Texas, USA) (78). Site-directed mutagenesis was used to add a stop codon after the SV40NLS. The NLSs of TDP43 (residues 82–98) and FUS (residues 495–526) were PCR-amplified using primers (see Supplemental Methods: Primers), digested with XbaI and BglII, and cloned into the corresponding sites in EYFP2-SV40NLS-NES. The NLS of MATR3 (residues 583-602) was purchased as a Geneblock from Integrated DNA Technologies, digested with XbaI and BglII, and cloned into the corresponding sites in EYFP2-SV40NLS-NES. DLK-pEGFPN1 was a gift from Gareth Thomas (Shriners Hospitals Pediatric Research Center, Center for Neurorehabilitation and Neural Repair, Temple University School of Medicine, Philadelphia, Pennsylvania, USA; and Department of Anatomy and Cell Biology, Temple University School of Medicine, Philadelphia, Pennsylvania, USA) (79). The coding sequence of DLK-GFP was amplified with the stop codon, and an N-terminal 3× Flag tag was added using primers by 2 rounds of PCR and cloned into the pGW1 plasmid between the cloning sites KpnI and SalI. All plasmids were verified by Sanger sequencing. See the Supplemental Methods for additional details.

### Data analysis.

For analysis of immunocytochemistry images, ROIs were generated using CVAT (68) or manually drawn in ImageJ. Whole cell masks were based on the staining for endogenous NLK or FLAG-tagged proteins (whole cell); nuclear masks were generated from Hoechst-stained nuclei (Supplemental Figure 2). Cytoplasmic ROIs were defined as the remaining mask after the subtraction of the nuclear ROI from the whole-cell ROI. Speckle and paraspeckle masks were generated with custom CellProfiler pipelines (Supplemental Figure 5, C and D). Nucleoli were manually counted by a researcher without knowledge of the condition. For IHC images, a pathologist selected each neuron, manually drew an ROI, and annotated the TDP43 status (pathology or no pathology) using custom Fiji scripts. Images were deconvolved to quantitate NLK signal, which was then normalized within each slide, again using custom Fiji scripts.

### Statistics.

Statistical analysis was performed with GraphPad Prism 9 and graphs were generated with R. Statistical information, including mean and statistical significance values, is indicated in the figures and figure legends. At least 3 biological replicates were used per experiment. Data were considered statistically significant for *P* values less than 0.05.

### Study approval.

Skin samples for iPSC creation were collected and deidentified in collaboration with the Michigan Institute for Clinical and Health Research (UL1TR000433) through an IRB-approved protocol (HUM00028826).

### Data availability.

Raw data for this manuscript are in the Supporting Data Values file associated with this manuscript. FASTQ data reported in this paper (Figure 12B) are available in the NCBI’s Sequence Read Archive under PRJNA925944 (https://www.ncbi.nlm.nih.gov/sra/PRJNA925944). All correspondence regarding the materials and data presented in this paper should be addressed to the corresponding author.

## Author contributions

MEB and SJB designed the study. EP and MEB designed experiments with SJB. MEB and EP collected data for most experimental studies, analyzed the data, assembled the figures, and wrote the manuscript. MEB and EP performed immunocytochemistry. JJM and MH assisted with data collection and analysis. MEB performed survival assays in iNeurons, cultured rat neurons expressing NLK, and wrote custom Fiji scripts for quantitation of immunocytochemistry and dual IHC. EP made YFP reporter constructs, supervised dual IHC (with the IHC core), and selected motor neurons for quantitation. FBG aided in conception of the study and aided in development of poly GP and poly GR ELISAs. GK developed and performed poly GP and poly GR ELISAs. LG and MM contributed to mbOrg conception, development, and data analysis. HHS and CAC conceived of and performed DLK survival assay and immunocytochemistry. HHS performed survival assays in cultured rat neuron DLK. MK and SC cultured mbOrgs and performed RNA-Seq and qRT-PCR. EMU contributed to the original concept and development of mbOrgs and provided RNA-Seq data from mbOrgs. XL isolated and cultured rat cortical neurons. EMHT derived iPSC lines and developed protocols for their differentiation. JW developed original code for neuron segmentation and image processing. SJB provided resources, funding, and conceptual input for experiments and supervised the research. All authors were involved throughout the research process; agreed among themselves regarding roles and responsibilities; and contributed to the review, editing, and approval of the manuscript.

## Supplementary Material

Supplemental data

Unedited blot and gel images

Supporting data values

## Version 1 06/24/2025

In-Press Preview

## Version 2 09/02/2025

Electronic publication
