# Supplementary material for: Nemo-like kinase disrupts nuclear import and drives TDP43 mislocalization in ALS
Source: J Clin Invest. 2025 Jun 24;135(17):e188138. doi: 10.1172/JCI188138 (PMC12404744; doi:10.1172/JCI188138)
Supplement: Supplemental data [file jci-135-188138-s017.pdf]

Nemo-like kinase disrupts nuclear import and drives TDP43 mislocalization in ALS

Michael E. Bekier II[1#], Emile Pinarbasi[1,2#], Gopinath Krishnan[3], Jack J. Mesojedec[1], Madelaine Hurley[1], Harisankar Harikumar Sheela[4], Catherine Collins[5], Layla Ghaffari[63], Martina de Majo[63], Erik Ullian[74], Mark Koontz[63], Sarah Coleman[63], Xingli Li[1], Elizabeth M. H. Tank[1], Jacob Waksmacki[1], Fen-Biao Gao[3], Sami Barmada[1*]

[1]Department of Neurology, University of Michigan, Ann Arbor, MI, 48109, United States

[2]Department of Pathology, Michigan Medicine, University of Michigan, Ann Arbor, MI, 48109, United States

[3]Frontotemporal Dementia Research Center, RNA Therapeutics Institute, University of Massachusetts Chan Medical School, Worcester, MA 01605

[4]Department of Molecular, Cellular, and Developmental Biology, University of Michigan, Ann Arbor, MI, 48109

[5]Department of Neurosciences, Case Western Reserve University School of Medicine, Cleveland, OH, 44106

[6]Synapticure, Chicago, IL, 60612

[7]Department of Ophthalmology, University of California San Francisco, San Francisco, CA, 94143

# contributed equally

*Correspondence to Sami Barmada: 109 Zina Pitcher Place, Ann Arbor, MI, 48019; 734-764-8425; sbarmada@umich.edu

Supplemental Material

Supplemental figures and legends

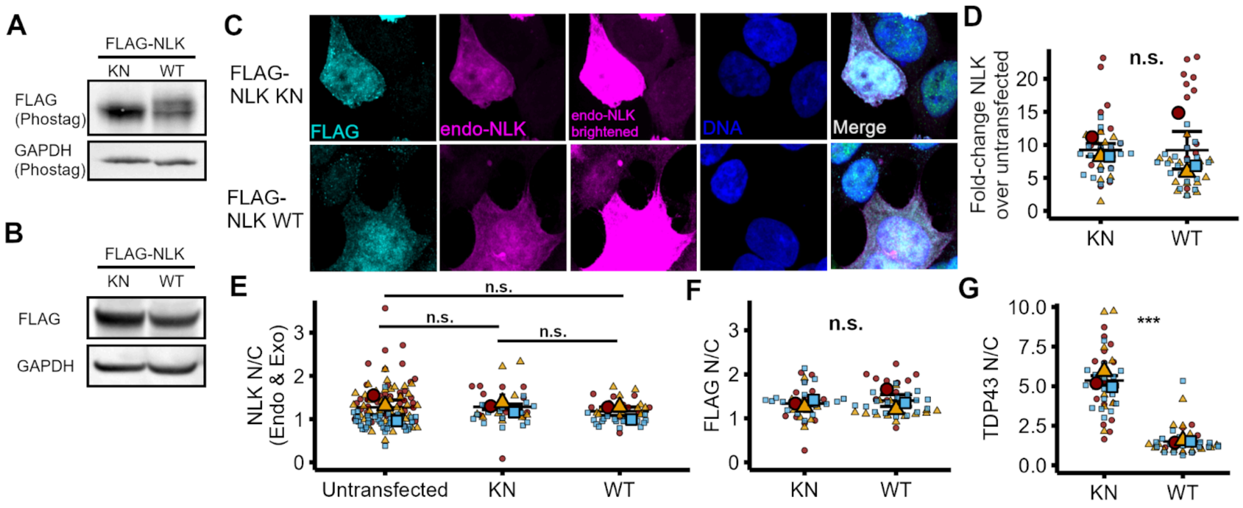

Supplemental Figure 1. Validation of FLAG-NLK plasmids and analysis of endogenous versus exogenous NLK localization and levels related to Figure 1.(**A**) Western blot of lysates from HEK293 cells transfected with FLAG-NLK constructs, run on PhosTag acrylamide SDS-PAGE, showing a phosphorylation-dependent mobility shift in FLAG-NLK WT but not FLAG-NLK KN, consistent with kinase activity and autophosphorylation. (**B**) Standard SDS-PAGE Western blot of the same lysates as in (**A**), demonstrating equivalent expression of FLAG-NLK WT and KN.

(**C**) Immunofluorescence using antibodies against endogenous NLK (magenta) and exogenous FLAG-tagged NLK (cyan) reveals similar subcellular localization to both the nucleus and cytoplasm. Nuclei were stained with Hoechst (blue). (**D**) Quantification of fold-change in total NLK levels (based on anti-NLK staining) in FLAG-NLK-expressing cells relative to untransfected controls. (**E**) Quantification of nuclear-cytoplasmic ratio (N/C) of total NLK (anti-NLK antibody) in untransfected and FLAG-NLK-expressing cells. (**F**) Quantification of N/C ratio of exogenous

FLAG-NLK, showing comparable nuclear and cytoplasmic localization for both WT and KN forms. (**G**) Quantification of TDP-43 N/C ratio in FLAG-positive cells from the same population as in (**F**), showing TDP-43 mislocalization in cells expressing FLAG-NLK WT but not KN.

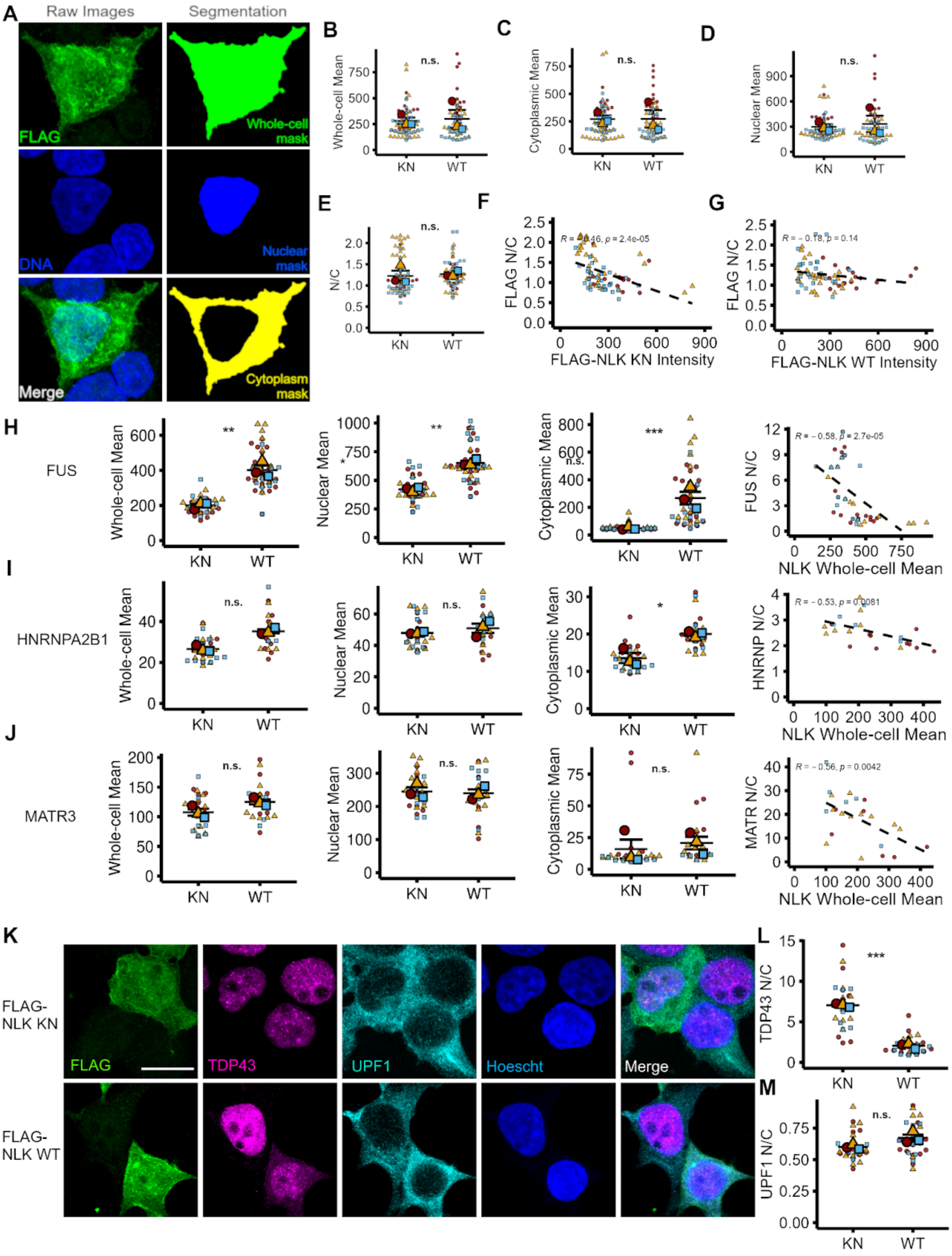
A
Raw Images
Segmentation
FLAG
Whole-cell mask
DNA
Nuclear mask
Merge
Cytoplasm mask
B
Whole-cell Mean
n.s.
KN
WT
C
Cytoplasmic Mean
n.s.
D
Nuclear Mean
n.s.
E
N/C
F
FLAG N/C
FLAG-NLK KN Intensity
G
FLAG-NLK WT Intensity
H
FUS
**
***
FUS N/C
NLK Whole-cell Mean
I
HNRNPA2B1
*
HNRNP N/C
J
MATR3
MATR N/C
K
FLAG-NLK KN
FLAG-NLK WT
FLAG
TDP43
UPF1
Hoescht
Merge
L
TDP43 N/C
M
UPF1 N/C

Supplemental Figure 2. Supporting quantification from Figure 1. (**A**) Representative images illustrating how nuclear and cytoplasmic compartments are defined in HEK293 cells. FLAG immunostaining is used to generate a whole-cell mask, while Hoechst DNA staining defines the nuclear mask. The cytoplasmic mask is derived by subtracting the nuclear area from the whole-cell area, enabling differentiation between nuclear and cytoplasmic compartments. (**B–G**) Supporting quantification for Figure 1A–E, showing FLAG-NLK whole-cell, cytoplasmic, and nuclear intensities, as well as nuclear-cytoplasmic ratio (N/C). Scatterplots show the relationship between FLAG-NLK whole-cell intensity and corresponding N/C ratios. (**H–J**) Supporting quantification for Figure 1G–L, showing whole-cell, cytoplasmic, and nuclear intensities of the indicated endogenous proteins, along with their N/C ratios. Scatterplots illustrate the relationship between whole-cell intensity and N/C ratio. (**K**) HEK293 cells were transfected with plasmids encoding either FLAG-NLK KN or FLAG-NLK WT, followed by immunofluorescence using antibodies against FLAG (green), TDP-43 (magenta), and UPF1 (cyan). Nuclei were stained with Hoechst (blue). Scale bar = 10 μm. (**L**) Superplot of UPF1 N/C ratios from images in (K). Line = mean; error bar = standard error. No significant difference observed (unpaired t-test with Welch's correction). (**M**) Superplot of TDP-43 N/C ratios from the same cells as in (K), showing a significant reduction with NLK WT expression.

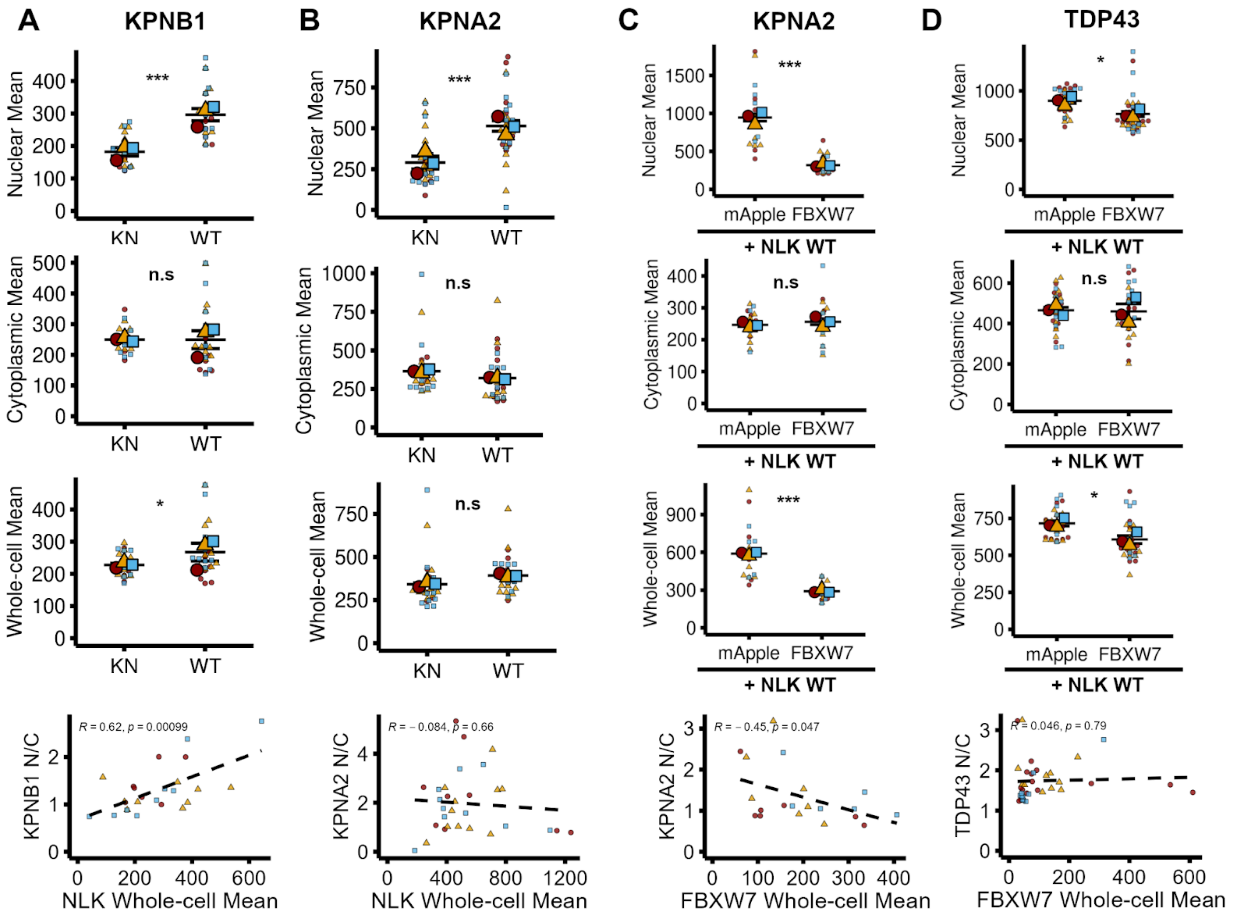

Supplemental Figure 3. Supporting quantitation from Figure 3. (**A**) Superplot of KPNB1 nuclear, cytoplasmic, and whole-cell intensities following overexpression of FLAG-NLK KN or FLAG-NLK WT (related to Figure 3A). Statistical significance is indicated as follows in all sub-panels: n.s. = not significant, *p < 0.05, **p < 0.01, ***p < 0.001 (unpaired t-test with Welch's correction). (**B**) Superplot of KPNA2 nuclear, cytoplasmic, and whole-cell intensities following overexpression of FLAG-NLK KN or FLAG-NLK WT (related to Figure 3B). Line = mean; error bar = standard error. (**C**) Superplot of KPNA2 nuclear, cytoplasmic, and whole-cell intensities following co-expression of FLAG-NLK WT with either mApple or V5-FBXW7 (related to Figure 3C). Line = mean; error bar = standard error. (**D**) Superplot of TDP-43 nuclear, cytoplasmic, and whole-cell intensities following co-expression of FLAG-NLK WT with either mApple or V5-FBXW7 (related to Figure 3D). Line = mean; error bar = standard error.

Supplemental Figure 4

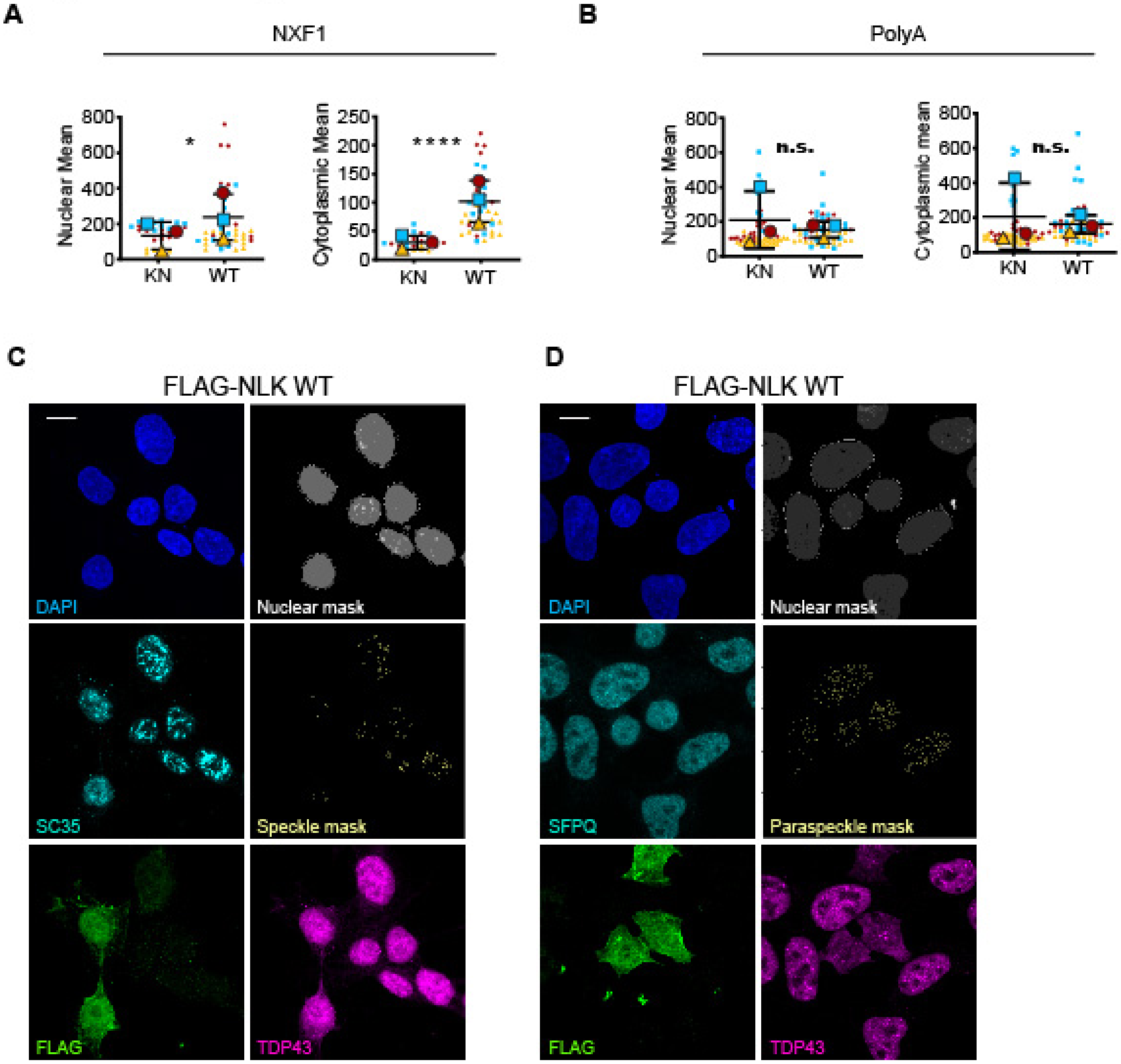

Supplemental Figure 4. Additional quantifications from Figure 5. (**A**) Superplot of NXF1 nuclear and cytoplasmic levels after NLK KN or NLK WT overexpression related to Figure 5B. *p<0.05, ****p<0.0001. (unpaired T-test with Welch's correction). (**B**) Superplot of PolyA nuclear and cytoplasmic levels after NLK KN or NLK WT overexpression related to Figure 5C. n.s., not significant as determined by unpaired T-test with Welch's correction. (**C**) Representative image of HEK cells transfected with plasmids encoding either FLAG-NLK KN or FLAG-NLK WT followed by immunofluorescence for FLAG (green), TDP43 (magenta) and markers of speckles

(SC-35; cyan); DNA was stained with Hoescht (blue). Nuclear masks (Hoescht signal) and speckle masks (SC35) were generated using CellProfiler. (**D**) Representative image of HEK293 cells transfected with plasmids encoding either FLAG-NLK KN or FLAG-NLK WT followed by immunofluorescence for FLAG (green), TDP43 (magenta), and markers of paraspeckles (SFPQ, cyan); DNA was stained with Hoescht. Nuclear masks (Hoescht signal) and paraspeckle masks (SFPQ) were generated using CellProfiler.

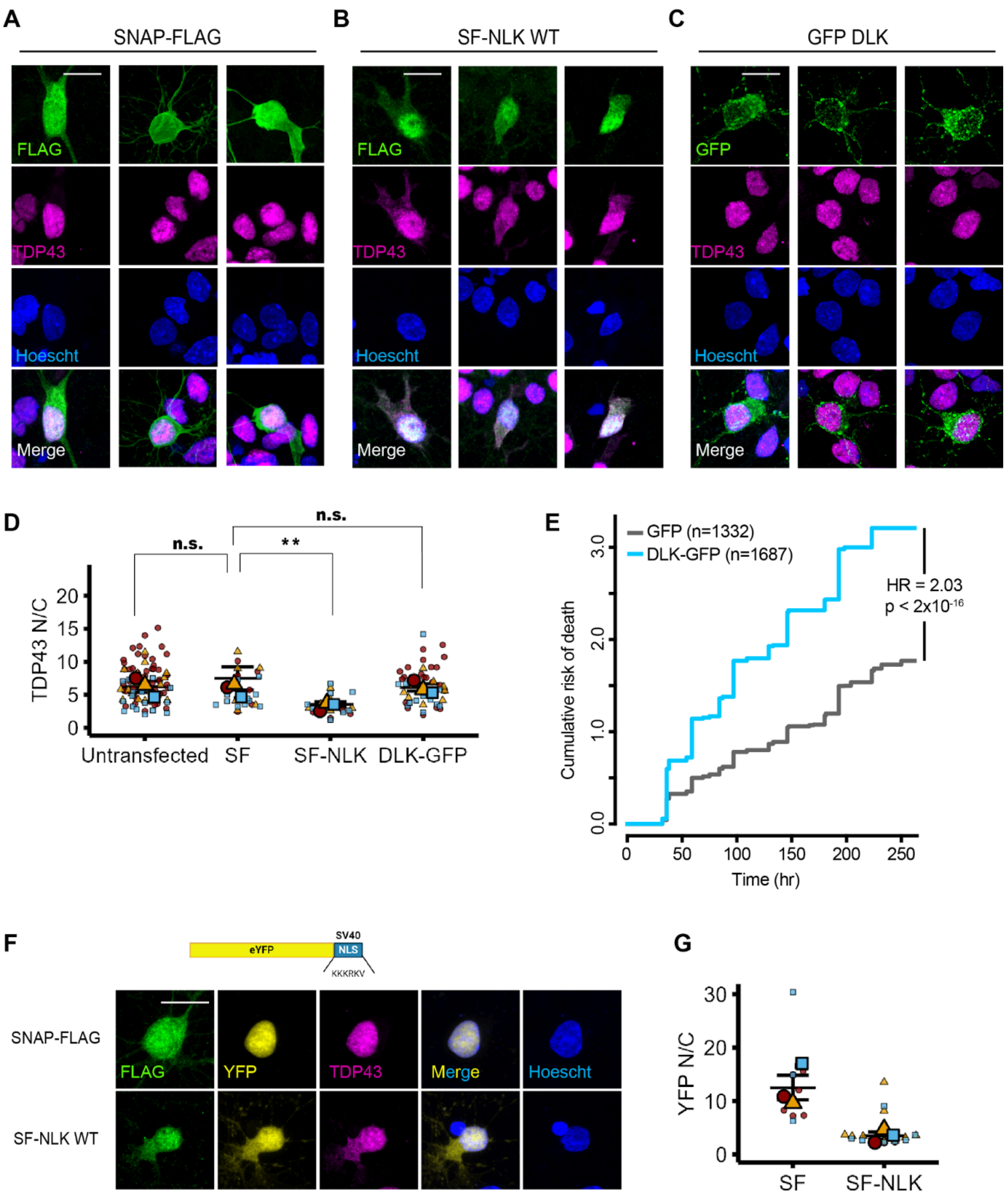

Supplemental Figure 5. Supplemental images and quantification supporting Figure 5.

(**A-B**) Additional representative images of rodent primary cortical neurons transfected with either SNAP-FLAG (SF; negative-control) or SNAP-FLAG-NLK (SF-NLK) followed by

immunofluorescence using antibodies against FLAG (green) and TDP43 (magenta). DNA was stained with Hoechst (blue) (**C**) Rodent primary cortical neurons were transfected with DLK-GFP followed by immunofluorescence using antibodies TDP43 (magenta) and direct visualization of tagged protein (green). DNA was stained with Hoechst (blue). (**D**) Superplots of N/C ratio of TDP43 in untransfected rodent primary cortical neurons and those transfected with SNAP-FLAG (SF; negative-control), SNAP-FLAG-NLK (SF-NLK), and DLK-GFP, corresponding to representative images in A-C. n.s. not significant. ** $p<0.01$ (Dunnett's T3 multiple comparisons test). (**E**) Cumulative Hazard plot showing the relative risk of death in neurons expressing either GFP or DLK-GFP, calculated by Cox proportional hazards analysis, stratified among 3 biological replicates. HR, hazard ratio. (**F**) Rodent primary cortical neurons were co-transfected with YFP-NLS^{SV40} and either SNAP-FLAG (SF; negative-control) or SNAP-FLAG-NLK (SF-NLK) followed by immunofluorescence using antibodies against FLAG (green) and TDP43 (magenta). DNA was stained with Hoechst (blue). Scale bar = 10 μm. (**G**) Superplots of N/C ratio of TDP43 in cells corresponding to (F). *** $p<0.001$ (unpaired t test with Welch's correction).

A

	ALS - 1	ALS - 2	ALS - 3	ALS - 4	Ctrl - 1	Ctrl - 2	Ctrl - 3	Ctrl - 4
Male/female (M/F)	M	F	M	M	M	F	M	F
Age (y)	64	76	64	51	61	78	72	64
Clinical diagnosis	ALS with FTLD	ALS	ALS	ALS	Early-onset AD	Probable AD	Lewy Body Dementia	HD
Neuropathological diagnosis	ALS<br>PSP	ALS	ALS	ALS	ADNC, high	ADNC, int	ADNC, high<br>LATE-NC, stage 1	HD<br>ADNC, low

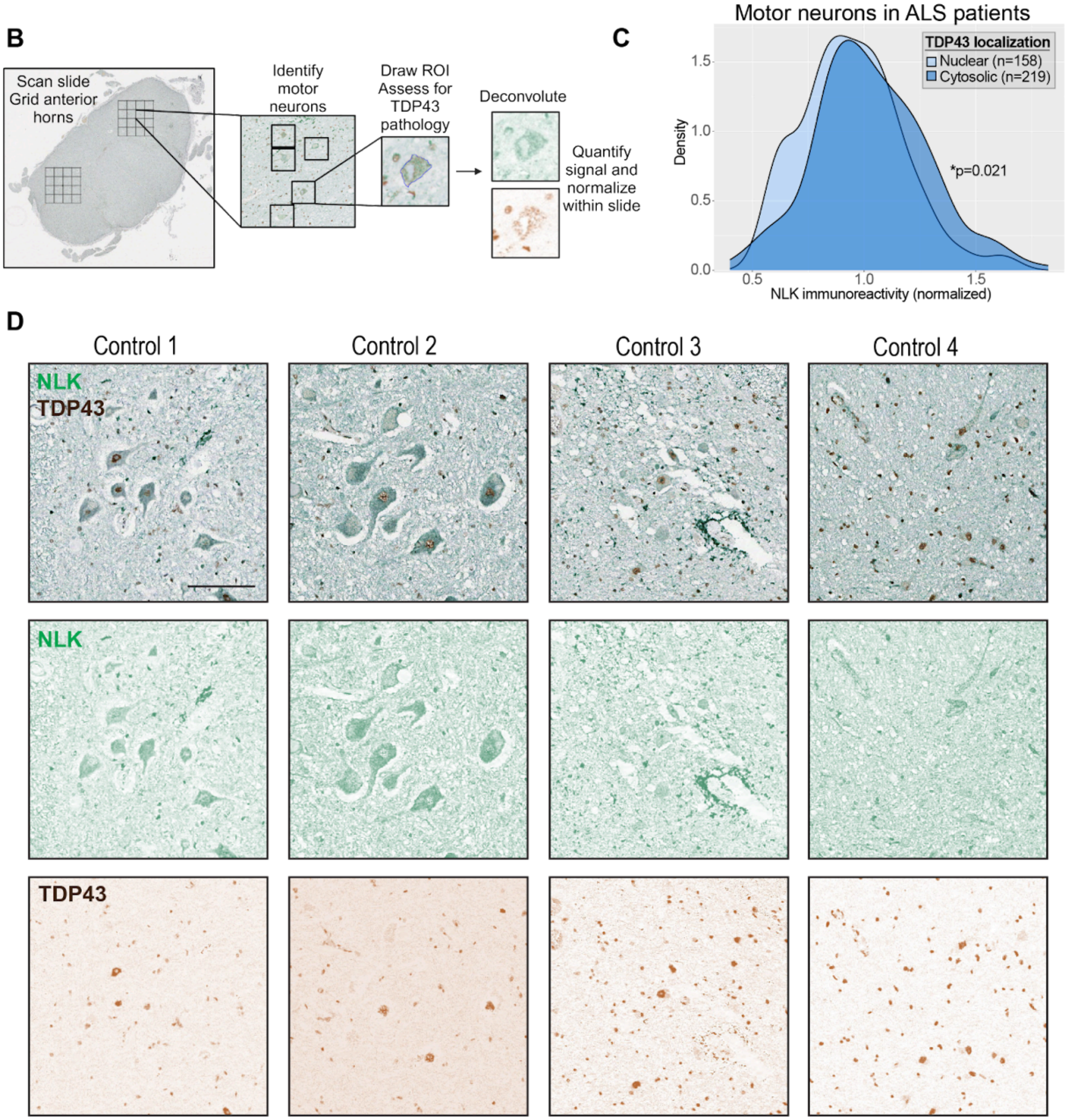

Supplemental Figure 6. NLK/TDP43 dual immunohistochemistry in patient tissue. (**A**) Patient characteristics. Ctrl: control. ALS: amyotrophic lateral sclerosis. HD: Huntington's disease. AD: Alzheimer's disease. ADNC: Alzheimer disease neuropathologic change. LATE-NC: Limbic predominant age-related TDP43 encephalopathy neuropathologic change. (**B**) Schematic showing workflow for quantification of dual immunohistochemistry. (**C**) Density plot depicting the change in NLK immunoreactivity in motor neurons with and without TDP43 pathology, corresponding to images in 7E. * $p<0.05$ by 2-sided Kolmogorov Smirnov test. (**D**) Dual immunohistochemistry for NLK and TDP43, performed on spinal cord tissue from four control patients without spinal cord pathology. Scale bar = 100 μm.

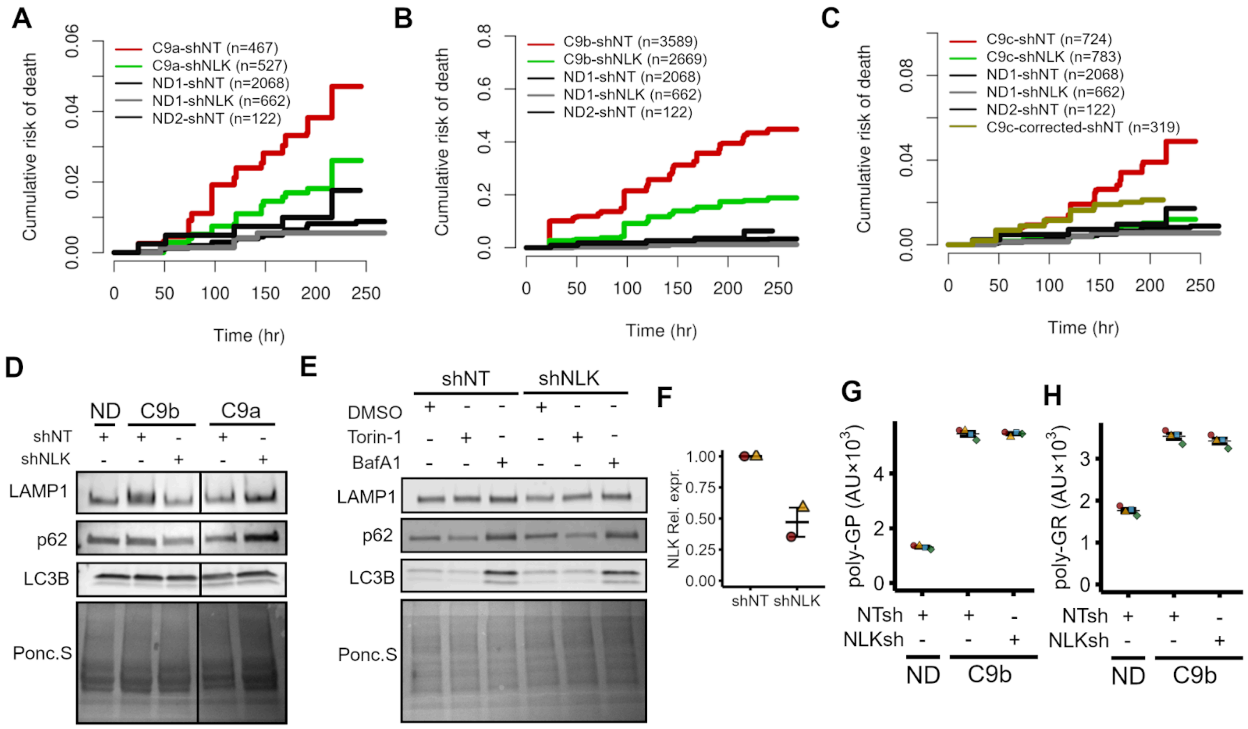

Supplemental Figure 7. Supporting quantification and data for Figure 7. (**A-C**) Cumulative hazard plots showing the relative risk of death in neurons from three independent C9orf72 patient-derived iPSC lines (C9a, C9b, and C9c), following transduction with lentivirus encoding either non-targeting (scramble) or NLK-directed shRNA. Each is compared to neurons from two non-disease (ND) control iPSC lines. (**D**) Western blot analysis of lysates from ND, C9a, and C9b iNeuron cultures showing no consistent changes in LAMP1, p62, or LC3B levels following NLK knockdown. (**E**) Western blot analysis of HEK293 cells stably expressing either non-targeting (NT) or NLK-directed shRNA showing no changes in LAMP1, p62, or LC3B upon NLK knockdown. (**F**) qPCR showing reduced NLK mRNA levels in HEK293 cells stably expressing NLK shRNA compared to NT controls. (**G,H**) Poly-GP and poly-GR ELISA measurements in C9b neurons after transduction with shNT or shNLK, showing no significant change in dipeptide repeat levels following NLK knockdown.

Supplemental Methods:

iPSC lines:

	**ID**	**Onset age**	**Biopsy age**	**Gender**	**Onset**	**Notes**
**C9orf72-fALS**	C9b (883)	49	51	M	Lumbar	C9orf72 mutation +
	C9a (312)	52	54	M	Lumbar	C9orf72 mutation +
	C9c (CS52)	46	47	M	Lumbar	C9orf72 mutation +
**TDP43-fALS**	TDP43 (M337V)		54	F		M337V and C-terminal Dendra2 inserted into ND1 (1021) by CRISPR/Cas9
	TDP43 (WT)		54	F		C-terminal Dendra2 inserted intoND1 (1021) by CRISPR/Cas9
**Control**	ND1 (1021)		54	F		Healthy
	ND2 (746)		58	M		Healthy
	C9c-corrected	46	47	M	Lumbar	C9orf72 mutation removed from CS52i by CRISPR/Cas9

137 **Antibodies:**

	Vendor	Catalog #	Concentration for ICC	Conc. for WB
Flag	AbCam	ab1170	1:200	1:1000
TDP43	R&D	MAB7778	1:200	1:1000
TDP43	Proteintech	10782-2-AP	1:200	
FUS	Sigma	HPA008784	1:200	
HNRNPA2B1	Proteintech	14813-1-AP	1:100	
MATR3	Abcam	ab281927	1:200	
V5	Abcam	ab53418	1:200	
KPNA2	Proteintech	10819-1-AP	1:200	1:500
KPNB1	Abcam	AB2811	1:200	1:500
Ran	BD Transduction labs	610340	1:400	
RanGAP	Abcam	AB92360	1:200	1:500
RanBP2	Abcam	AB64276	1:200	1:1000
Mab414	Abcam	AB24609	1:200	

NXF1	Abcam	AB129160	1:200	
SC-35	Sigma	S4045-.2ML	1:200	
SFPQ	Proteintech	15585-1-AP	1:200	
Nucleophosmin	Millipore	MABE937	1:200	
NLK	Abcam	Ab97642	1:200	
UPF1	Abcam	Ab133564	1:200	
GAPDH	Millipore	MAB374		1:1000

Plasmids

Plasmid	Source
FLAG-NLK-WT	Gift from T. Ishitani (66)
FLAG-NLK KN (K155M)	Gift from T. Ishitani (66)
EYFP2-SV40NLS-NES	Gift from Yuh Min Chook (67)
EYFP2-SV40NLS	This paper
EYFP2-TDPNLS	This paper
EYFP2-FUSNLS	This paper
EYFP-MATR3NLS	This paper

pGW1-TDP43(2F/L)-EGFP	Flores et al 2019 (36)
FUGW-SNAP-FLAG	This paper
FUGW-SNAP-FLAG-NLK	This paper
DLK-GFP	This paper

Primers

Amplify TDP43 NLS	For: ctcagatctcgaAAAAGAAAAATGGAT<br><br>Rev: ttatctagagatTCTTTTCACTTTCAC
Amplify FUS NLS	For: tcagatctCGAGGGGGCCGGGGT<br><br>Rev: tatctagaATACGGCCTCTCCCT
Mutagenesis: introduce stop codon into EYFP2-SV40NLS-NES	For:<br>ctcgaccaaaaaagaagagaaaggtataagctttagccttgaaa<br><br>Rev: tttcaaggctaaagcttatacctttctcttctttttggtcgag
Primers used to clone pGW1 DLK-GFP	Forward primer to amplify FLAG-DLK:<br>atcatgacatcgattacaaggatgacgatgacaagctcGCCTGCCTCCATGAAACCCGAA

	Forward primer to amplify Kpn1-FLAG:<br>ggataggtacCATGgactacaaagaccatgacggtgattataaag<br>atcatgacatcgatt<br><br>Reverse primer to amplify Sal1-EGFP:<br>tgcaggtcgaCTTACTTGTACAGCTCGTCCATGCCG<br>AG
qPCR NLK	For: GGGTCCTCATAAACAGCCATC<br><br>Rev: AGACCAACATCCTGCAAAGG
qPCR GAPDH	For: GGG GTC ATT GAT GGC AAC AAT A<br><br>Rev: ATG GGC AAG GTG AAG GTC G
