# Supplementary material for: Nemo-like kinase disrupts nuclear import and drives TDP43 mislocalization in ALS
Source: J Clin Invest. 2025 Jun 24;135(17):e188138. doi: 10.1172/JCI188138 (PMC12404744; doi:10.1172/JCI188138)
Supplement: Unedited blot and gel images [file jci-135-188138-s018.pdf]

Full western blot images

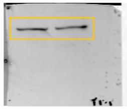

Figure 3A TDP43 WB

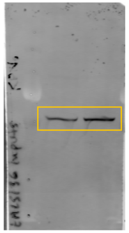

Figure 3A KPNA2 WB

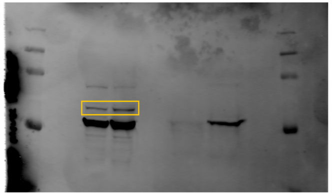

Figure 3A KPNB1 WB

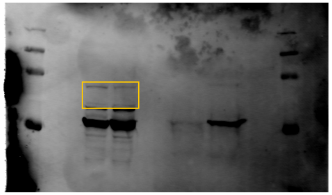

Figure 3A RanGAP1 WB

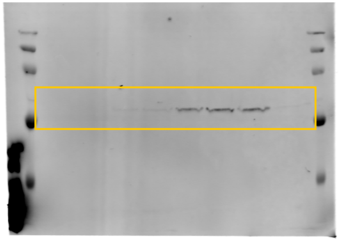

Figure 3B FLAG WB

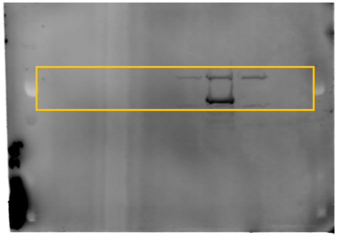

Figure 3B RanGAP1 WB

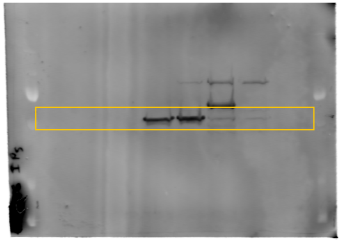

Figure 3B KPNA2 WB

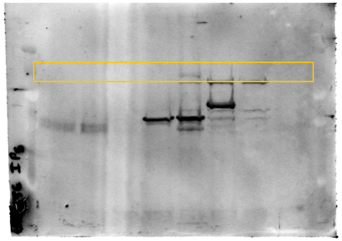

Figure 3B KPNB1

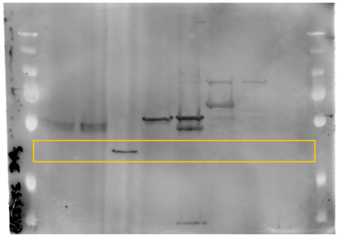

Figure 3B TDP43 WB

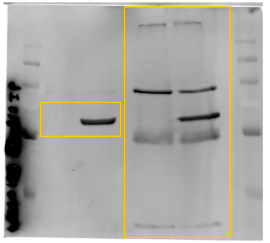

Figure 3A FLAG (left)
Figure 3C RanBP2, RanGAP1, FLAG WB (

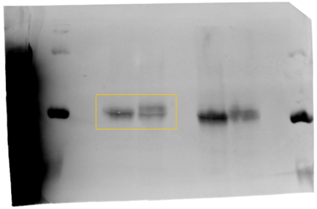

Figure S1A
FLAG phostag WB

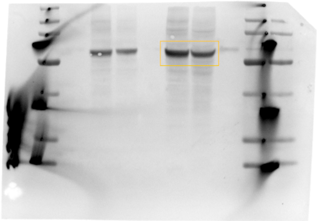

Figure S1B FLAG WB

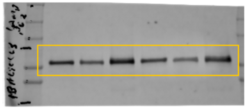

Figure S8E p62 WB

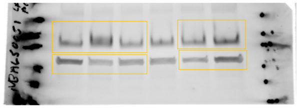

Figure S8D LAMP1 WB (top)
Figure S8D p62 WB (bottom)

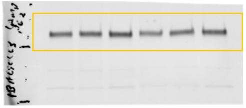

Figure S8E LAMP1 WB

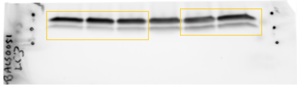

Figure S8D LC3B WB

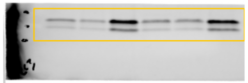

Figure S8E LC3B WB

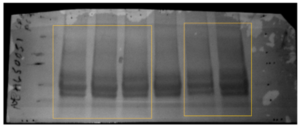

Figure S8D PonceauS

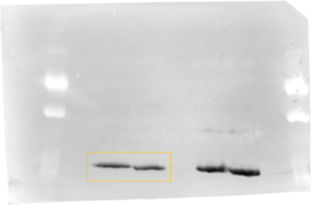

Figure S1A GAPDH WB

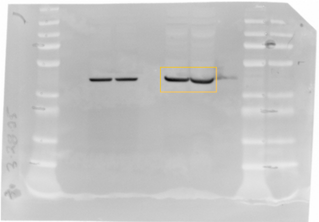

Figure S1B GAPDH WB

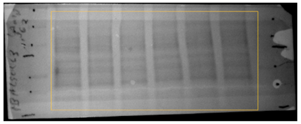

Figure S8E PonceauS
